# A unique form of collective epithelial migration is crucial for tissue fusion in the secondary palate and can overcome loss of epithelial apoptosis

**DOI:** 10.1242/dev.200181

**Published:** 2022-05-26

**Authors:** Teng Teng, Camilla S. Teng, Vesa Kaartinen, Jeffrey O. Bush

**Affiliations:** 1 Department of Cell and Tissue Biology, University of California San Francisco, San Francisco, CA 94143, USA; 2 Program in Craniofacial Biology, University of California San Francisco, San Francisco, CA 94143, USA; 3 Institute for Human Genetics, University of California San Francisco, San Francisco, CA 94143, USA; 4 Eli and Edythe Broad Center of Regeneration Medicine and Stem Cell Research, University of California San Francisco, San Francisco, CA 94143, USA; 5 Department of Biologic and Materials Sciences, University of Michigan School of Dentistry, Ann Arbor, MI 48109, USA

**Keywords:** Palate, Collective cell migration, Apoptosis, Cell extrusion, Tgfβ, Cleft palate, Cleft lip, Craniofacial, Live imaging, Fusion, Actomyosin, Cell adhesion, Non-muscle myosin II, NMIIA, Mouse

## Abstract

Tissue fusion frequently requires the removal of an epithelium that intervenes distinct primordia to form one continuous structure. In the mammalian secondary palate, a midline epithelial seam (MES) forms between two palatal shelves and must be removed to allow mesenchymal confluence. Abundant apoptosis and cell extrusion support their importance in MES removal. However, genetically disrupting the intrinsic apoptotic regulators BAX and BAK within the MES results in complete loss of cell death and cell extrusion, but successful removal of the MES. Novel static- and live-imaging approaches reveal that the MES is removed through streaming migration of epithelial trails and islands to reach the oral and nasal epithelial surfaces. Epithelial trail cells that express the basal epithelial marker ΔNp63 begin to express periderm markers, suggesting that migration is concomitant with differentiation. Live imaging reveals anisotropic actomyosin contractility within epithelial trails, and genetic ablation of actomyosin contractility results in dispersion of epithelial collectives and failure of normal MES migration. These findings demonstrate redundancy between cellular mechanisms of morphogenesis, and reveal a crucial and unique form of collective epithelial migration during tissue fusion.

## Abstract (precis)

**Summary:** Multiple cellular processes mediate secondary palate fusion, including a unique form of streaming collective epithelial migration driven by pulsatile actomyosin contractility.

## INTRODUCTION

Tissue fusion is a complex morphogenetic process that occurs in diverse developmental contexts and organisms (Hashimoto et al., 2015; Hayes and Solon, 2017; Ray and Niswander, 2012; Rothenberg and Fernandez-Gonzalez, 2019). This process is crucial in the development of multiple midfacial structures, including the upper lip, primary palate, secondary palate and nasal septum (Cox, 2004; Ji et al., 2020; Jiang et al., 2006; Lan et al., 2015). The mammalian palate separates the oral and nasal cavities and comprises the primary and secondary palate. The primary palate forms a small anterior part of the hard, bony palate. The anterior part of the secondary palate consists of bony structures, and the posterior part of the secondary palate consists of the muscular soft palate. Disruptions in the development of these structures can result in orofacial clefts, a common class of congenital anomaly that requires surgical intervention after birth and can have long-term health implications. In the USA, approximately 1 in 2800 babies is born with a cleft lip without a cleft palate, 1 in 1700 babies is born with cleft palate without a cleft lip, and 1 in 1600 babies is born with a cleft lip and cleft palate (Mai et al., 2019). Given that clefts of the lip and secondary palate occur together more often than they do individually, their etiologies are sometimes conflated, although their development is spatiotemporally distinct. The upper lip and primary palate form through fusion between the medial nasal process and the maxillary and lateral nasal processes at embryonic day (E) 10.5-11.5 of mouse development, whereas final closure of the secondary palate involves fusion between the secondary palatal shelves at around E14.5-15.5 of mouse development. Although these tissue fusion events are independent, some common general principles appear to be shared. For example, tissue fusion in each of these contexts involves tight regulation of the initiation of epithelial adhesion followed by the formation and removal of an intervening epithelial seam. Given their unique topologies, the cellular mechanisms are likely to be different. Tissue fusion has been most extensively investigated for the secondary palate, but the mechanisms remain mysterious at the cellular level.

The mammalian secondary palatal shelves arise as bilateral anlagen of the maxillary processes and undergo outgrowth, elevation above the tongue, horizontal growth toward the midline and, ultimately, fusion with one another to form the intact roof of the mouth (Ferguson, 1988; Lan et al., 2015). The palatal epithelium making contact is called the medial edge epithelium (MEE) and comprises an outer layer of Keratin 6a (Krt6a)-expressing squamous periderm cells that protect the palatal shelves against premature fusion and an inner layer of basal, cuboidal cells expressing the transcription factor ΔNp63 (Richardson et al., 2014; Fitchett and Hay, 1989; Hammond et al., 2019). After the palatal shelves meet (E14.5 in mice), the opposing layers of MEE form an intervening midline epithelial seam (MES) that must be removed in order to achieve confluence of the underlying mesenchyme. Failure to complete secondary palate fusion can result in an overt or a submucous cleft palate, in which the oral mucosa is superficially intact but fails to form a continuous underlying structure. Nasolabial or median palatal epithelial cysts can also result from the enclavement of epithelium during failed embryonic palate fusion (Cinberg and Solomon, 1979; Kuriloff, 1987; Queiroz et al., 2011).

The current predominant model holds that removal of the MES and completion of secondary palate fusion is driven mostly by epithelial apoptosis (Carette and Ferguson, 1992; Clarke, 1990; Cuervo and Covarrubias, 2004; Cuervo et al., 2002; Hammond et al., 2019; Huang et al., 2011; Jin and Ding, 2006; Kim et al., 2015; Richardson et al., 2017; Shapiro and Sweney, 1969; Vaziri Sani et al., 2005; Xu et al., 2008). Apoptosis plays crucial roles in many aspects of organogenesis, and its regulation can be categorized into intrinsic and extrinsic pathways (Green, 1998). The intrinsic pathway is activated from within the cell and involves the activation of proapoptotic BCL-2 family proteins BAX, BAK (BAK1) and BOK, which regulate the mitochondrial release of cytochrome *c* and formation of a protein complex called the apoptosome, which includes the protein APAF1. The apoptosome initiates a caspase cascade that culminates in the proteolytic cleavage and activation of the effector caspases 3, 6 and 7 (Fuchs and Steller, 2011). The extrinsic pathway is initiated by the activation of death receptors, which recruit multiple adaptors ultimately converging on caspase 8 to activate the same effector caspases. Common to both apoptotic mechanisms, effector caspases orchestrate destruction of the cell by cleavage of vital proteins (Danial and Korsmeyer, 2004; Nagata, 1997). Other forms of cell death include necrosis and necroptosis; although these do not involve the same cleaved caspase cascade, they ultimately converge on similar outcomes, such as DNA fragmentation (Vanden Berghe et al., 2010).

Numerous reports observed significant apoptosis in the MES during fusion stages, and apoptosis correlates with the capacity for secondary palate fusion in some mutants, such as those with perturbed TGFβ3 signaling (AlMegbel and Shuler, 2020; Cuervo and Covarrubias, 2004; Huang et al., 2011; Iwata et al., 2011; Ke et al., 2019; Lan et al., 2015; Martínez-Álvarez et al., 2000; Shapiro and Sweney, 1969). In several tissue culture studies, pan-caspase inhibitor treatment resulted in reduced palate fusion (Cuervo and Covarrubias, 2004; Cuervo et al., 2002; Huang et al., 2011). Genetic disruption of apoptosis in *Bok*^−/−^*;Bax*^−/−^*;Bak*^−/−^ mutants resulted in a cleft palate phenotype, although loss of apoptosis in this model was not restricted to the epithelium and these mutants also exhibited a cleft face phenotype that developmentally precedes the fusion step of secondary palatogenesis (Ke et al., 2018). Moreover, conflicting results have been reported; pan-caspase inhibitor treatment did not disrupt palate fusion in some studies (Takahara et al., 2004) and loss of APAF1 has been reported to result in cleft palate by some researchers (Cecconi et al., 1998; Honarpour et al., 2000) but not by others (Jin and Ding, 2006). However, APAF1-independent mechanisms of cell death exist (Nagasaka et al., 2010), leaving the role of the intrinsic apoptotic pathway in secondary palate fusion uncertain. Extrinsic apoptosis mediated by the Fas ligand (FasL) has also been proposed to drive epithelial cell death during secondary palate fusion, and FasL expression was lost upon genetic perturbation of TGFβ signaling in the palatal epithelium in mouse embryos (Huang et al., 2011; Xu et al., 2020). In addition, extensive cell extrusion has been observed during removal of the intervening MES, although whether extruding cells were all apoptotic or also included live cells was not clear (Kim et al., 2015; Schüpbach and Schroeder, 1983; Schüpbach et al., 1983).

Epithelial migration has also been proposed to contribute to the elimination of MES cells (Carette and Ferguson, 1992; Jin and Ding, 2006; Kim et al., 2015; Logan and Benson, 2020; Richardson et al., 2017). It has been reported that TGFβ3 signaling downregulates ΔNp63 to cause basal cell cycle arrest and enable periderm cell migration to the oral and nasal aspects of the palatal shelves, which is thought to reveal the underlying basal epithelium and facilitate cell death (Cuervo and Covarrubias, 2004; Hammond et al., 2019; Richardson et al., 2017). Using live imaging, we previously proposed a mechanism of convergent displacement to explain the observed movement of epithelial cells to the oral surface; however, our understanding of this cell migratory mechanism was technically limited by the achievable depth of imaging with that approach, and its relative functional significance remains unproven (Kim et al., 2015).

Here, we present previously unreported approaches for the study of secondary palate fusion, which we use to interrogate functionally the cellular behaviors that drive this process. Consistent with previous reports, we found that apoptosis is abundant within the MES during its removal, supporting its involvement with normal secondary palate development; however, genetically abolishing cell death in the epithelium only resulted in a slight delay of MES removal. Instead, through a combination of novel static- and live-imaging approaches, we uncovered a surprising progression of collective epithelial cell migratory patterns. Small breaks in the MES consolidate into an interconnected network of epithelial trails connecting to the oral and nasal surfaces, and epithelial islands that undergo apoptosis or migrate through the mesenchyme. Whereas adherens junctions couple epithelial trail cells during migration, filamentous actin is anisotropically enriched at the edges of trails. Actomyosin contractility is crucial for this unique form of epithelial migration, and its disruption resulted in the dissolution of epithelial collectives and failure to complete secondary palatal shelf fusion. These results provide insights into the cellular mechanisms driving secondary palate fusion and indicate that multiple cellular processes mediate this crucial morphogenetic event.

## RESULTS

### A new imaging approach shows that apoptosis is not required for MES removal during palate fusion

**Fig. 1.**
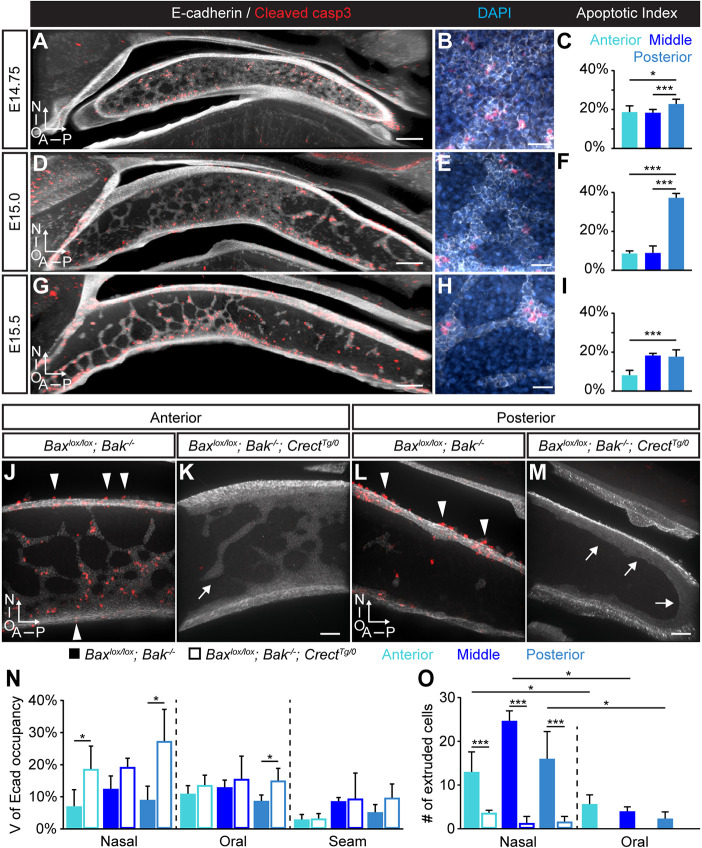
**Apoptosis is abundant in the MES but its loss does not prevent MES removal.** (A-I) Patterns of MES removal and apoptosis at E14.75 (A-C), E15.0 (D-F) and E15.5 (G-I). (A,B,D,E,G,H) 3D-rendered views of sagittal sections of wild-type mouse secondary palate immunostained for E-cadherin (white) and cleaved caspase 3 (red) at low (A,D,G) and high (B,E,H) magnification reveals that, at E14.75 (A,B), the E-cadherin-expressing MES is organized in a mostly continuous sheet with small breaks in the anterior MES. (D,E) At E15.0, larger and more numerous gaps in the MES leave a network of epithelial trails connecting to the oral and nasal epithelial surfaces. (G,H) By E15.5, the remaining MES is organized into a series of interconnecting trails, with some epithelial islands broken away from epithelial trails. At each of the above stages, cleaved caspase 3 immunostaining reveals the abundance of MES apoptosis and DAPI (blue) reveals mesenchymal nuclei interspersed between MES epithelial trail nuclei (E,H). (C,F,I) Quantification of apoptosis across distinct anteroposterior regions during fusion. Histograms show the apoptotic index, which is the ratio of the number of apoptotic MES cells to the total number of MES cells in the entire secondary palate at each stage. Column height represents the mean of the ratio for *n*=3 per stage. (J-M) 3D-rendered images of E15.5 mid-anterior (J,K) and posterior regions (L,M) of *Bax^lox/lox^; Bak*^−/−^ control (J,L) and *Bax^lox/lox^; Bak*^−/−^*; Crect^Tg/0^* (K,M) sagittal thick sections immunostained for E-cadherin (white) and cleaved caspase 3 (red). Arrowheads indicate apoptotic cell extrusion events in *Bax^lox/lox^; Bak*^−/−^ embryos, which are lost in *Bax^lox/lox^; Bak*^−/−^*; Crect^Tg/0^* embryos, which also exhibit thickened MES trails that are more slowly removed (arrows in K,M). (N) Quantification of volume of E-cadherin occupancy reveals the extended retention of MES. (O) Quantification of cell extrusion in indicated palatal regions. Column height represents the mean from *n*=3 per genotype. Error bars represent s.e.m.; *, *P*<0.03; ***, *P*<0.001 determined by unpaired Student's *t*-tests. Scale bars: 20 µm in A,D,G; 50 µm in J-M; 200 µm in B,E,H; N, nasal surface; O, oral surface.

To characterize cell behaviors during secondary palate fusion, we first aimed to better visualize the MES. Given that traditionally used coronal sections provide a view of the MES at one anteroposterior position, we established a sagittal thick-sectioning technique to enable visualization of the MES in its entirety (Fig. S1A-C). Static imaging of thick sections of the MES immunostained for E-cadherin (cadherin 1) at progressive stages of MES removal revealed a surprising pattern of MES clearance (Fig. 1A-I). Small breaks in the epithelium appeared immediately before E14.75 and widened over time to give the appearance of a web-like network of trails connecting to the oral and nasal surface epithelium (Fig. 1A,D,G). Small epithelial islands, which appeared to have separated from the trails, were apparent at E15.5 (Fig. 1G). Epithelial trails and islands were surrounded by mesenchymal cells that could be visualized by immunostaining for vimentin (Fig. S1C). At each of these stages, cleaved caspase 3 immunostaining revealed that 10-40% of MES cells undergo apoptosis, consistent with the prevailing understanding that apoptotic death is the ultimate fate of a substantial proportion of MES cells during normal secondary palate fusion (Fig. 1A,C,D,F,G,I) (Cuervo and Covarrubias, 2004; Cuervo et al., 2002). We found that a greater proportion of MES cells in the posterior palate undergo apoptosis compared with those in more-anterior positions (Fig. 1C,F,I). At E15.0, the number of apoptotic cells deep within the MES was similar to the number at the oral and nasal surfaces (known as epithelial triangles). By E15.5, more apoptotic cells were observed within the epithelial triangles at the oral and nasal surfaces than within trails. However, normalization of apoptotic cell number to the volume of E-cadherin-expressing cells revealed relatively greater apoptosis outside of the epithelial triangles (Fig. S2E,F). Apoptosis was highly localized to the MES, with exceedingly few apoptotic cells detected in the surrounding mesenchyme (Fig. S2G,H). This previously unreported imaging perspective enables renewed investigation of the patterns of MES removal during secondary palate development.

We next sought to study the function of apoptosis within the MES without confounding effects of earlier malformations, using the *Crect* craniofacial ectoderm Cre driver to disrupt apoptosis within the MES (Reid et al., 2011). Although this Cre driver has been used to study lip and primary palate fusion (Lee et al., 2020; Losa et al., 2018; Reid et al., 2011), we verified by using the *R26 [Gt(ROSA)26Sor]^mTmG^* reporter (Muzumdar et al., 2007) that it also mediates highly efficient recombination within the secondary palate epithelium. Although we observed highly efficient and specific recombination within the epithelium and MES along most of the secondary palate of *Crect^Tg/0^; R26^mTmG^*^/+^ embryos (Fig. S3A-C,E-G), the far-posterior mesenchyme also exhibited GFP reporter expression (Fig. S3D,H). *In situ* hybridization by RNAscope revealed an abundance of *Cre* transcript expression within the mesenchymal cells of this region (Fig. S3J), indicating that the far-posterior mesenchymal *Crect^Tg/0^; R26^mTmG^*^/+^ signal was likely attributable to unexpected *Crect* activity (Fig. S3I,J). Nevertheless, given that *Crect* activity was highly specific throughout most of the palatal epithelium, we used it to disrupt *Bax* specifically in the MES of *Bak* mutant mice. Whereas *Bax^lox/lox^; Bak*^−/−^ embryos exhibited extensive apoptotic cell death similar to wild type (Fig. S4A), cell death was completely lost in the MES of *Bax^lox/lox^; Bak*^−/−^*; Crect^Tg/0^* E15.5 embryos, assayed by both TUNEL analysis and cleaved caspase 3 immunostaining (Fig. 1J-M; Fig. S4B,C; Fig. S5). Quantifying the volume of E-cadherin-expressing MES cells at E15.5 revealed that, whereas cell death loss did not prevent secondary palate fusion, it did appear to change the pattern and timing of MES cell removal (Fig. 1J-N). This effect was most substantial within the posterior secondary palate, in which the MES was retained near the oral, posterior and nasal surfaces of the posterior palate (Fig. 1L-N), consistent with the greater amount of MES apoptosis in the posterior (Fig. 1C,F,I). Although we cannot rule out that reduced MES clearance in the posterior palate is attributable to the loss of mesenchymal apoptosis in this region, very few mesenchymal cells exhibit apoptosis normally (Fig. S2G,H), strongly suggesting this is not the case. Furthermore, we additionally observed that epithelial trails were consistently wider throughout the palate, suggesting that more cells were undergoing alternative removal processes upon loss of apoptosis (Fig. 1J,K; Fig. S4J-L). Cell extrusion was abundant on the oral and nasal surfaces of the secondary palates of controls (Fig. 1J,L,O), but nearly completely lost in *Bax^lox/lox^; Bak*^−/−^*; Crect^Tg/0^* E15.5 embryos (Fig. 1K,M,O), indicating that the apoptotic form of cell extrusion predominates, but that its loss does not result in failure of MES removal. Together, these results indicate that most cell death within the MES is attributable to the intrinsic apoptotic pathway, that cell extrusion is largely apoptotic, and that upon loss of both of these cellular mechanisms, secondary palate fusion and MES clearance complete successfully. Indeed, histological analysis confirmed that an intact secondary palate was observed at E17.5 in *Bax^lox/lox^; Bak*^−/−^*; Crect^Tg/0^* embryos (Fig. S4D-I). Therefore, although these mechanisms may normally contribute to MES removal, their loss can be overcome by other cellular mechanisms.

Epithelial-to-mesenchymal transition (EMT) has also been proposed to contribute to MES removal, but genetic lineage tracing did not reveal a contribution of MES cells to the underlying mesenchyme (Fitchett and Hay, 1989; Gritli-Linde, 2007; Vaziri Sani et al., 2005; Xu et al., 2006). Nevertheless, we wondered whether loss of apoptosis might result in a compensatory increase in EMT to clear MES cells. We performed genetic lineage tracing of the epithelium in *Bax^lox/lox^; Bak*^−/−^*; Crect^Tg/0^; R26^mTmG^*^/+^ and control mice and did not observe any GFP^+^Ecad(Cdh1)^−^ cells throughout most of the palate, indicating that compensatory EMT is not at play (Fig. S4A,C). We did detect GFP-expressing cells in the most posterior palatal shelf mesenchyme in both genotypes, consistent with posterior mesenchymal activity of *Crect* (Fig. S3D,H,J; Fig. S5B,D). These data indicate no compensatory EMT in most of the secondary palate, but our ability to detect EMT in the far-posterior palate was obfuscated by the mesenchymal activity of the *Crect* allele in this region. Also, *Crect^Tg/0^* mediated sporadic ectopic activity in around 15% of embryos. In *Crect^Tg/0^; R26^mTmG^*^/+^ embryos, this was discernible by scattered ubiquitous GFP expression; *Bax^lox/lox^; Bak*^−/−^*; Crect^Tg/0^; R26^mTmG^*^/+^ embryos exhibited vascular defects only when ectopic GFP recombination was observed, and these embryos were excluded from analysis. This activity profile will be an important consideration in future studies that make use of this nonetheless valuable mouse line (Reid et al., 2011).

### A unique form of collective epithelial migration drives secondary palate fusion

**Fig. 2.**
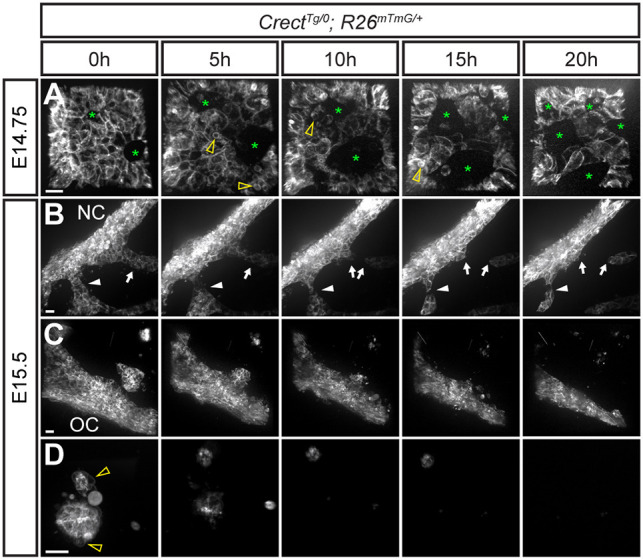
**A novel live imaging approach reveals patterns of collective epithelial migration in MES removal.** Confocal live imaging of EGFP in *Crect^Tg/0^; R26^mTmG^*^/+^ E14.75 (*n*=4) and E15.5 (*n*=5) embryos in various regions of the secondary palate reveals cell behaviors during MES removal. (A) Beginning from E14.75, the MES ‘sheet’ exhibits small breaks, which enlarge over time (asterisks). Arrowheads point to membrane blebbing. See also Movie 1. (B) Live imaging beginning at E15.5 revealed cell behaviors associated with the clearance of MES trails. An example of an MES cell trail moving through the unmarked mesenchyme toward the nasal surface as an epithelial collective (arrowhead); trails often break to form smaller trails or epithelial islands (arrows). See also Movie 2. (C) An example of an epithelial island close to the oral surface as it coalesces into the oral surface epithelium. See also Movie 3. (D) MES cells in an island far from the nasal or oral surfaces undergoing apoptosis. Arrowheads point to membrane blebbing as the island progressively shrinks and disappears. See also Movie 4. Scale bars: 15 µm. NC, nasal cavity; OC, oral cavity.

The patterns of epithelial removal described above suggested that collective cell migration may play a central role. We previously published a system for *ex vivo* live imaging of secondary palate fusion, which used confocal microscopy to image whole secondary palatal shelf explant cultures from the oral side of the MES (Kim et al., 2015, 2017). This approach was limited by the achievable depth of imaging, because only the most superficial cells at the epithelial triangles could be visualized. Therefore, we performed *ex vivo* live imaging of sagittal thick sections to observe MES removal in the secondary palate in *Crect^Tg/0^; R26^mTmG^*^/+^ embryos. We initiated imaging at multiple time points between E14.75 and E15.5 in order to gain a comprehensive understanding of epithelial behaviors during MES removal. First, by live imaging the mid-anterior MES for 20 h beginning at E14.75, we observed initial small breaks in the epithelium that enlarged as surrounding epithelial cells underwent rearrangements, breaking and re-establishing epithelial junctions until coalescing into a network of trails similar to what we described from our static imaging at E15.5 (Fig. 2A; Movie 1). We noticed extensive MES cell blebbing, consistent with the occurrence of apoptosis (Movie 1). Live imaging beginning at E15.5 revealed that epithelial trails streamed continuously toward the surface epithelium (Fig. 2B; Movie 2). During their migration, epithelial trails often broke into smaller cell collectives, ostensibly forming the previously described epithelial islands (Fig. 2B; Movie 2). When epithelial islands were close to the surface epithelium, they ultimately contacted and coalesced with the oral or nasal surface epithelium (Fig. 2C; Movie 3). However, epithelial islands that were deep within the secondary palatal shelves exhibited apoptotic bodies and membrane blebbing as they progressively disappeared through apparent cell death over the course of imaging (Fig. 2D; Movie 4).

**Fig. 3.**
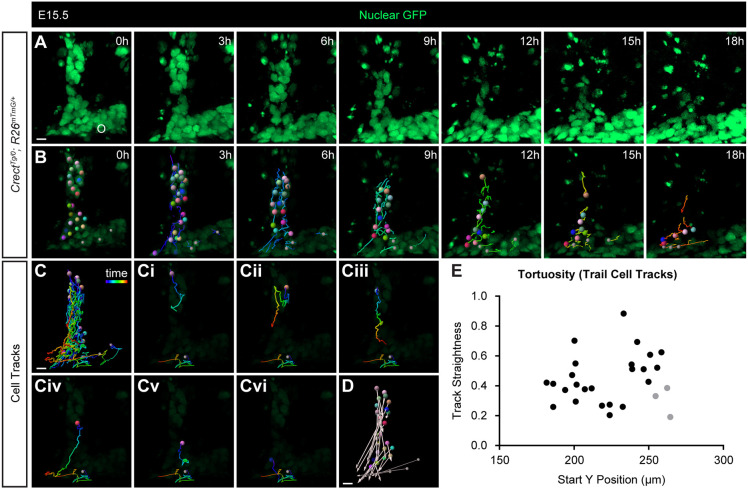
**Epithelial trail cells migrate collectively in MES removal.** (A,B) Time-lapse imaging of cells in an EGFP-labeled trail of an E15.5 *Crect^Tg/0^; R26^nTnG^*^/+^ embryo. Panels in B are the same as images in A with lowered brightness and overlaid with cell-tracking information. Individual cells are labeled with a colored sphere and tail indicating the path taken over the previous 3 h. See also Movie 5. Bright EGFP signals remaining in area adjacent to trail are likely apoptotic debris unconnected to trail cells. (C) Complete cell tracks of all cells from the trail and three cells from the oral epithelium (smaller gray spheres, as reference) indicate the entire length of the path taken by the cell over 18 h. (Ci-Cvi) A single cell track from the trail is shown with a single reference cell track in each panel as a representation of track patterns. (D) Vectors indicate the distance traveled by each cell, with spheres at the start point and arrowheads at the end point. (E) Track straightness (displacement/length) is plotted against the Y position of each cell at 0 h, with the axis origin at the top-left corner of the image. A total of ten trails from five embryos were imaged, and cells from one of two tracked trails using Imaris are represented in E. Scale bars: 10 µm.

**Fig. 4.**
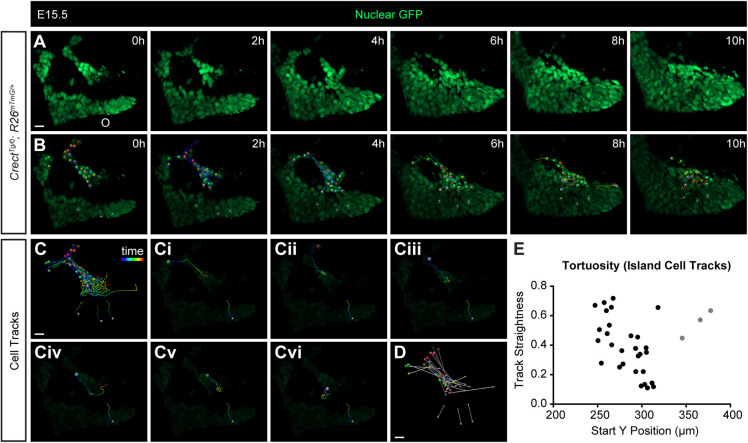
**Epithelial island cells migrate collectively in MES removal.** (A,B) Time-lapse imaging of cells in an EGFP-labeled island of an E15.5 *Crect^Tg/0^; R26^nTnG^*^/+^ embryo. Images in B are the same as images in A with lowered brightness and overlaid with cell-tracking information. Individual cells are labeled with a colored sphere and tail indicating the path taken over the previous 2 h. See also Movie 6. (C) Complete cell tracks of all cells from the island and three cells from the oral epithelium (smaller gray spheres, as reference) indicate the entire length of cell path taken over 10 h. (Ci-Cvi) A single cell track from the island is shown with a single reference cell track in each panel as a representation of track patterns. (D) Vectors indicate the distance traveled by each cell, with spheres at the start point and arrowheads at the end point. (E) Track straightness (displacement/length) is plotted against the Y position of each cell at 0 h, with the axis origin at the top-right corner of the image. A total of nine islands from four embryos were imaged, and cells from one trail were tracked using Imaris. Scale bars: 15 µm.

To determine how epithelial cells moved as a collective, we used the nuclear *R26^nTnG^* Cre reporter to track individual nuclei. Tracking individual cells of a trail from the middle palate revealed that, although some rearrangements occurred within epithelial streams, the nuclei moved collectively from positions deeper within the seam to the oral epithelium (Fig. 3; Movie 5). Although the paths of some cells in an epithelial trail were more tortuous than the paths of others, this was not dependent on the distance away from the oral epithelium, as measured by the Y position at the start of imaging (Fig. 3E). We also performed cell tracking of epithelial islands. Although many (*n*=5/5) epithelial islands appeared to resolve through apoptosis, individual cells of a larger island from the anterior palate exhibited collective movement toward the oral epithelium (Fig. 4; Movie 6). Individual cell tracks revealed similar tortuous paths to the epithelium (Fig. 4E), with cells deeper within the mesenchyme traversing a similar path to cells closer to the epithelium (Fig. 4C).

**Fig. 5.**
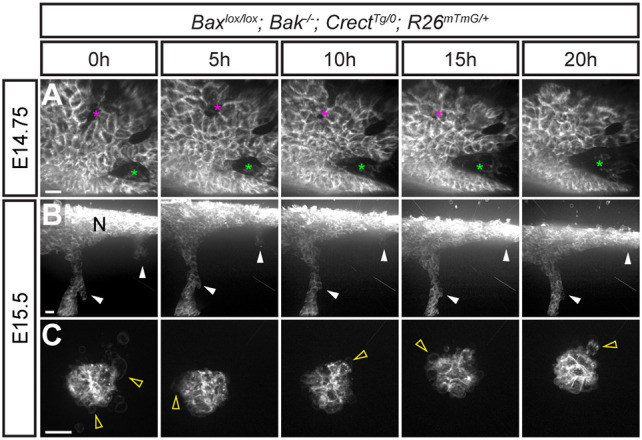
**Loss of apoptosis results in changes in MES cell removal but retention of collective epithelial cell migration.** (A-C) Confocal live sagittal section imaging of *Bax^lox/lox^; Bak*^−/−^*; Crect^Tg/0^; R26^mTmG^*^/+^ embryos at E14.75 (*n*=3) and E15.5 (*n*=3) reveals MES cell behaviors upon loss of apoptosis. (A) Over a 20-h imaging period beginning at E14.75, dynamic cell rearrangements within the MES epithelium result in the expansion of an epithelial gap (green asterisks) and the closure of another (pink asterisks). See also Movie 7. (B) MES cells migrate as collective trails through unmarked mesenchyme, resulting in MES cells being incorporated into the nasal surface epithelium (arrowheads, Gamma 1.5, see also Materials and Methods). See also Movie 8. (C) Islands found deep within the palatal shelves and surrounded by unmarked mesenchyme did not disappear over a 20-h imaging period, but did exhibit membrane blebbing (arrowheads). See also Movie 9. N, Nasal surface. Scale bars: 15 µm.

Given that the MES was successfully removed in the absence of cell death, but that patterns of epithelial removal were altered, we performed live imaging of *Bax^lox/lox^; Bak*^−/−^*; Crect^Tg/0^; R26^mTmG^*^/+^ embryos to observe whether the cell behaviors underlying MES removal were altered upon loss of apoptosis. Epithelial breakage and collective migration of epithelial trails in mutants appeared overtly similar to those in control embryos at E15.5 (Fig. 5A,B; Movies 7,8). However, as noted above, consistently thicker epithelial trails in the mutants implied that more cells were undergoing collective migration (Fig. 1J,K; Fig. S4J-L), which was also supported by the extended retention of MES cells in the posterior palate (Fig. 1J). In addition, we observed reclosure of some epithelial breaks that we did not see in wild-type embryos (Fig. 5A; Movie 7). Epithelial islands in mutants underwent cell blebbing similar to control but failed to shrink or disappear in the imaging timeframe (Fig. 5C; Movie 9). These persistent epithelial islands together with occasional recovery of epithelial breakage may account for the mildly altered epithelial removal seen in *Bax^lox/lox^; Bak*^−/−^*; Crect^Tg/0^* mutants. Overall, our results indicate that in the absence of apoptosis, MES cells retain collective epithelial migration behaviors that compensate for the loss of apoptotic contributions to MES removal.

### Krt6a/p63-expressing epithelial cells undergo collective migration

**Fig. 6.**
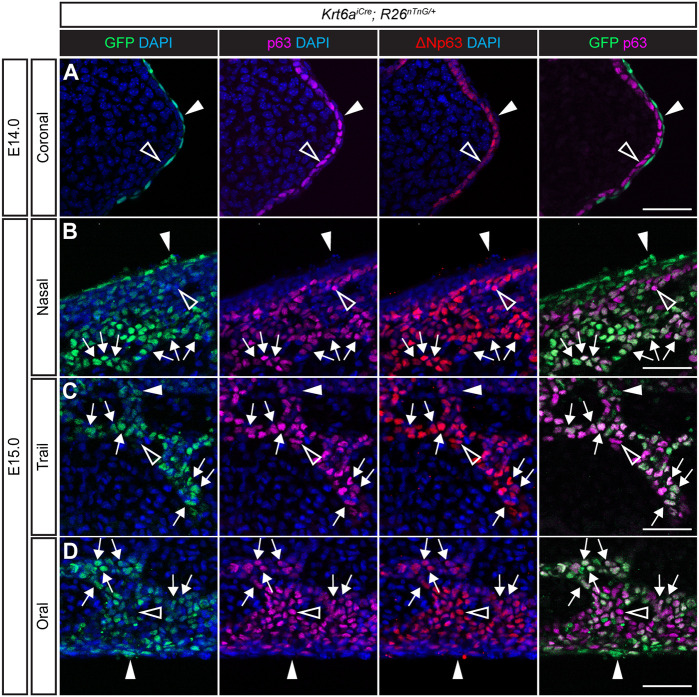
**Migrating MES cells exhibit a periderm-like Krt6a lineage while retaining basal marker p63.** (A) Immunostaining of coronal cryosections of E14.0 *Krt6a^iCre^; R26^nTnG^* embryos (*n*=3) for GFP (green), pan-p63 (magenta) and the ΔNp63 isoform (red) confirms Cre recombination activity specifically within the periderm prior to palatal shelf contact. (B-D) Optical slices of vibratome thick sagittal sections of E15.0 *Krt6a^iCre^; R26^nTnG^* embryos (*n*=5) reveals co-expression of the Krt6a lineage (GFP) with the basal markers p63 and ΔNp63 within most cells of the migratory MES trails. p63 expression was missing only from the GFP-expressing cells in ‘epithelial triangle’ regions close to the nasal (B) and oral (D) surfaces. Arrows point to GFP^+^p63^+^ cells. Filled arrowheads indicate GFP^+^p63^−^ cells, and open arrowheads point to GFP^−^p63^+^ cells. Scale bars: 25 µm.

Based on a previous report indicating that periderm cells undergo migration during palatal fusion (Richardson et al., 2017), and to discern between cell behaviors of peridermal versus basal MES cells, we generated a *Krt6a iCre* knock-in mouse line for tracking the migration of peridermal cells (see also the Materials and Methods). Crossing *Krt6a^iCre^* mice with the *R26^nTnG^* nuclear reporter allele confirmed that recombination was restricted to the periderm and was entirely non-overlapping with the ΔNp63-expressing basal epithelial cells prior to palatal shelf contact at E14.0 (Fig. 6A, arrowheads). Sagittal sections of the MES undergoing fusion at E15.0 showed that epithelial cells that were migrating (1) to the nasal surface (Fig. 6B), (2) within the medial aspect of the secondary palate (Fig. 6C) (3) or to the oral surface (Fig. 6D) mostly exhibited GFP expression reflecting a *Krt6a* lineage identity. *Krt6a^iCre^; R26^nTnG^* periderm lineage-positive cells that did not express p63 (Trp63) mostly resided within the ‘epithelial triangles’ of the nasal surface, suggesting that they may have completed migration before the basal epithelium did. Surprisingly, in migratory epithelial trails, we observed co-expression of the *Krt6a^iCre^; R26^nTnG^* periderm lineage marker together with p63. Given that our finding that p63 expression was retained within MES cells during fusion differs from what has been previously published (Richardson et al., 2014, 2017), we compared staining using an antibody recognizing pan-p63 with staining using an antibody specifically recognizing the ΔNp63 isoform. These antibodies exhibited identical staining patterns, suggesting that this discrepancy does not reflect a difference in isoform expression (Fig. 6A-D). Therefore, the abundance of Krt6a-lineage and p63 double-positive cells provides evidence that p63-expressing basal epithelial cells undergo migration to the oral and nasal surface concomitant with the basal epithelial initiation of expression of periderm marker Krt6a.

### Collective epithelial migration of the MES requires actomyosin contractility

**Fig. 7.**
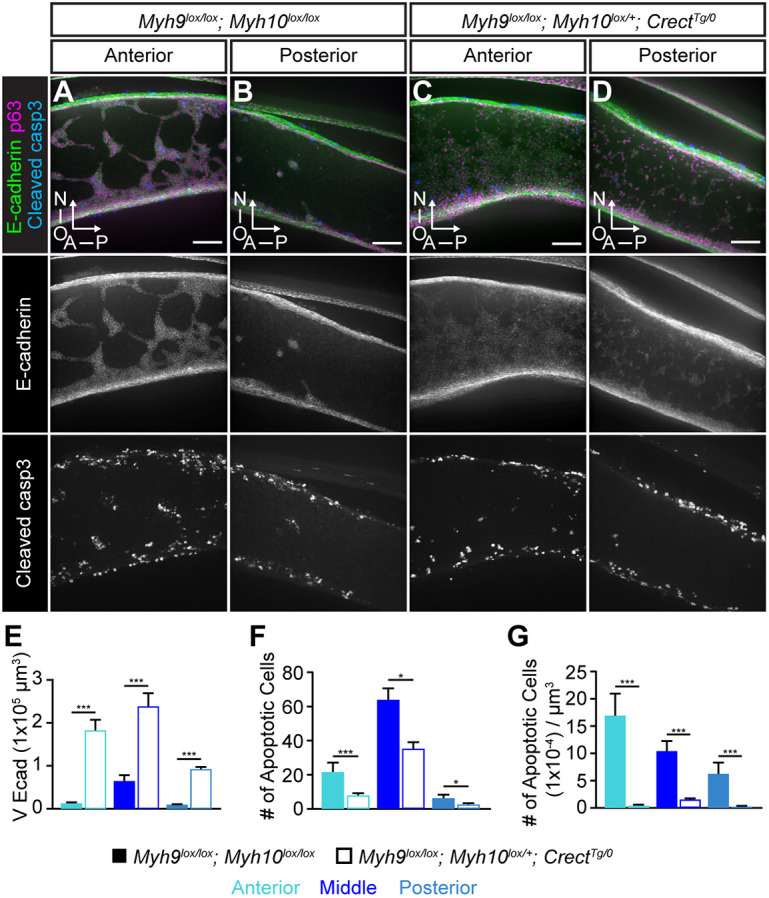
**Epithelial-specific loss of NMII results in failure to clear the MES**. Immunostained *Myh9^lox/lox^; Myh10^lox/lox^* control and *Myh9^lox/lox^; Myh10^lox^*^/+^*; Crect^Tg/0^* mutant sagittal sections of secondary palates. (A,B) In controls, E-cadherin-labeled MES cells are organized into collective trails and islands as they are removed from the palatal shelves. (C,D) In *Myh9^lox/lox^; Myh10^lox^*^/+^*; Crect^Tg/0^* embryos, abundant MES cells remain in the palate but are dispersed. (E) Quantification of E-cadherin expression volume (µm^3^) as a measure of MES removal, (F) number of apoptotic cells and (G) apoptotic cell number normalized by total E-cadherin volume in the anterior, middle and posterior palate of *Myh9^lox/lox^; Myh10^lox/lox^* controls and *Myh9^lox/lox^; Myh10^lox^*^/+^*; Crect^Tg/0^* mutants. Column height represents the mean from *n*=3. Error bars represent s.e.m.; *, *P*<0.03; ***, *P*<0.001 determined by unpaired Student's *t*-tests. Scale bars: 50 µm. A, anterior; N, nasal; O, oral; P, posterior.

Cell migration requires actomyosin contractility generated by non-muscle myosin; we previously discovered that the loss of non-muscle myosin isoforms NMIIA and NMIIB, encoded by *Myh9* and *Myh10*, respectively, results in a cleft posterior secondary palate (Kim et al., 2015; Vicente-Manzanares et al., 2009). Given that we could not recover *Myh9^lox/lox^; Myh10^lox/lox^; Crect^Tg/0^* embryos at palate fusion stages because of their cardiovascular phenotypes, we first examined loss of NMII function in *Myh9^lox/lox^; Myh10^lox^*^/+^*; Crect^Tg/0^* compound mutants using sagittal sectioning. We found that these embryos, which lacked NMIIA and had reduced NMIIB specifically in the MES, lost epithelial trail and island organization and exhibited a diffuse and dispersed epithelium at E15.5 (Fig. 7A-E). In addition to inappropriate retention of dispersed epithelium, *Myh9^lox/lox^; Myh10^lox^*^/+^*; Crect^Tg/0^* embryos also exhibited a decrease in the absolute number of apoptotic cells (Fig. 7F), and a dramatic decrease in apoptotic cells relative to the total amount of E-cadherin (Fig. 7G), suggesting that actomyosin contractility might be required to stimulate MES cell death. Given that E-cadherin was also reduced in *Myh9^lox/lox^; Myh10^lox^*^/+^*; Crect^Tg/0^* mutant palatal shelves, this quantification may be an underestimate of the volume of MES cells remaining and, therefore, also of the relative extent of reduced apoptosis. Analysis of individual *Myh9^lox/lox^; Crect^Tg/0^* embryos revealed a failure of MES removal that was similar to compound *Myh9^lox/lox^; Myh10^lox^*^/+^*; Crect^Tg/0^* embryos at E15.5, whereas *Myh9^lox^*^/+^*; Myh10^lox/lox^; Crect^Tg/0^* compound mutants looked similar to controls (Fig. S6), suggesting that NMIIA is the more crucial regulator. Although *Myh9^lox/lox^; Myh10^lox^*^/+^*; Crect^Tg/0^* embryos did not exhibit a cleft palate phenotype, an abundance of epithelial inclusions persisted in the secondary palate of *Myh9^lox/lox^; Crect^Tg/0^; R26^mTmG^*^/+^ mutants at E17.5 (Fig. S7), further supporting the importance of the NMIIA isoform in secondary palate fusion.

**Fig. 8.**
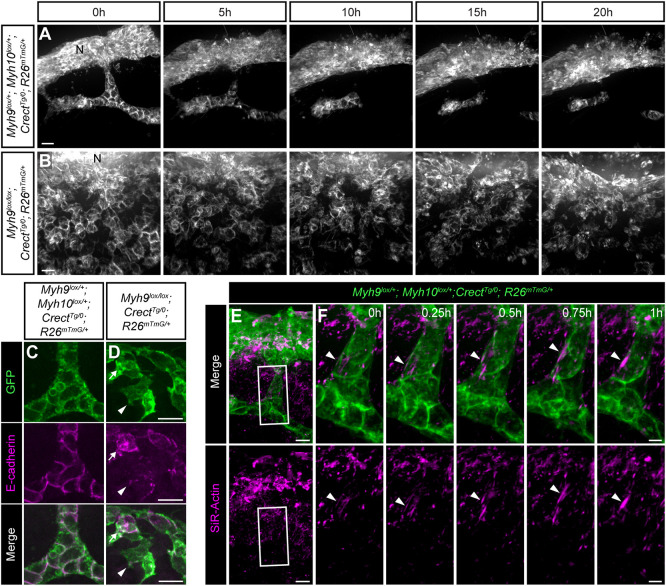
**Actomyosin contractility is required for collective epithelial organization and peristaltic movement of MES trails.** (A,B) Confocal live imaging of *Myh9^lox^*^/+^*; Myh10^lox^*^/+^*; Crect^Tg/0^; R26^mTmG^*^/+^ control (*n*=3) and *Myh9^lox/lox^; Crect^Tg/0^; R26^mTmG^*^/+^ mutant (*n*=3) sagittal sections at E15.5. (A) In controls, a trail of cells moves toward the nasal surface, where much of it is incorporated, but also breaks, resulting in island formation. See also Movie 10. (B) In the mutant, MES cells lose trail and island organization. Although they continue to move, they do not form trails and islands and do not clear the MES. See also Movie 11. (C,D) 3D rendering of immunostained MES trails at high magnification in E15.5 control (*n*=3) and *Myh9^lox/lox^; Crect^Tg/0^; R26^mTmG^*^/+^ (*n*=3) embryos reveals absence of junctional E-cadherin, an apparent reduction in E-cadherin protein levels (arrowheads) and a more protrusive shape upon loss of NMIIA. (E,F) Sagittal thick section confocal live imaging of E15.5 *Myh9*^*f/+*^*; Myh10*^*f/+*^*; Crect*^*Tg/0*^*; R26*^*mTmG/+*^ secondary palate (*n*=5). Frames shown represent a subset of the time lapse shown in A at a higher time-resolution. EGFP (green) labeling of MES cells and SiR-Actin (magenta) labeling of F-actin reveal anisotropic F-actin accumulation in epithelial trails. Pulsatile contractility of actomyosin is observed as the epithelial trail moves toward the nasal surface. See also Movie 12. (E) Low-magnification views of a single optical section. (F) 3D rendering of the boxed region in E at higher magnification. Arrowheads follow the movement of one F-actin filament. Scale bars: 5 µm in C,D,F; 10 μm in E; 15 µm in A,B. N, nasal surface.

To determine how loss of NMII activity affected epithelial cell behaviors underlying secondary palate fusion, we performed live imaging of *Myh9^lox/lox^; Crect^Tg/0^; R26^mTmG^*^/+^ embryos and compared them with controls with preserved NMII function (Fig. 8A,B). Whereas controls exhibited streaming collective epithelial migration (Fig. 8A; Movie 10), complete loss of NMIIA in *Myh9^lox/lox^; Crect^Tg/0^; R26^mTmG^*^/+^ mutants resulted in the complete loss of epithelial trail organization, wherein GFP^+^ MES-lineage cells that traveled in a disorderly fashion, had more protrusive shapes and were ultimately not cleared from the palatal shelf mesenchyme (Fig. 8B; Movie 11). At E15.5, highly ordered E-cadherin junctions are present between epithelial cells but not at the epithelial-mesenchymal cell interface in control MES cell trails (Fig. 8C; Fig. S6A,B). In contrast, GFP^+^ MES-lineage cells lacking NMIIA in mutants exhibited a dramatic loss of junctional E-cadherin localization, which also appeared reduced in abundance, possibly because of destabilization resulting from the loss of junctions (Fig. 8D; Fig. S6C-F). Interestingly, loss of epithelial architecture resulted in a highly protrusive, mesenchymal-like appearance of GFP^+^ MES-lineage cells in *Myh9^lox/lox^; Crect^Tg/0^; R26^mTmG^*^/+^ embryos, suggesting that NMIIA is required to maintain appropriate polarization of epithelial collectives (Fig. 8D; Fig. S6C-F) (Pandya et al., 2017). At the later timepoint of E17.5, GFP^+^ MES-lineage cells still retained E-cadherin, indicating that, in the absence of NMIIA, the MES loses epithelial organization and junctions, exhibits reduced E-cadherin and drastically changes its migratory cell behaviors; yet, these cells do not stably contribute to the palatal shelf mesenchyme by EMT in the majority of the secondary palate (Fig. S7A-D).

To better understand how NMII regulates collective epithelial migration, we performed live imaging of control (*Myh9*^*f/+*^*; Myh10*^*f/+*^*; Crect*^*Tg/0*^*; R26*^*mTmG/+*^) embryos treated with the SiR-Actin spirochrome dye to observe filamentous actin over the course of MES removal. We found that epithelial trails undergoing migration to the nasal and oral epithelium exhibited anisotropic distribution of filamentous actin cables at the epithelial-mesenchymal interface (Fig. 8E,F). As epithelial trails underwent collective movement toward the nasal and oral epithelium, these actomyosin cables contracted in a pulsatile fashion and appeared to move epithelial trails in a peristaltic fashion toward the oral and nasal edges, although precise manipulation of localized actomyosin contractility will be required to test this prediction functionally (Fig. 8E,F; Movie 12). Thus, these data reveal a unique form of collective epithelial migration that is responsible for MES clearance during secondary palate fusion.

## DISCUSSION

**Fig. 9.**
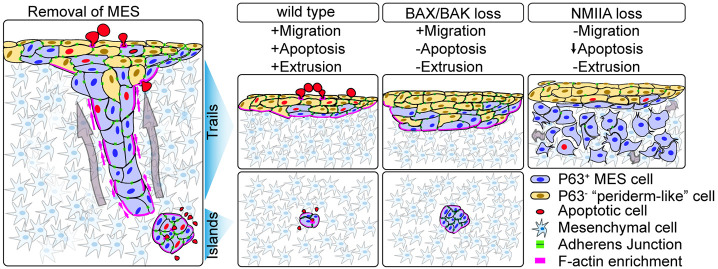
**Cell behaviors driving MES removal during secondary palate fusion.** Collective migration of MES cells occurs through the streaming migration of trails, which sometimes break into islands. We hypothesize that trails are removed through actomyosin contractility-dependent peristaltic collective movement, whereas islands are removed through either apoptosis or migration. Loss of actomyosin contractility through mutation of NMIIA results in dispersal of epithelial collectives and failure to clear the MES. Loss of function of BAX and BAK results in a loss of cell extrusion and apoptosis, causing changes in the pattern and timing of MES removal. Upon loss of apoptosis, epithelial islands do not shrink, but are still cleared from the palate, presumably by migration toward the oral and nasal surfaces; loss of apoptosis does not prevent completion of MES removal.

The cellular mechanisms driving secondary palate fusion have been investigated for more than three decades, leading to the thought that apoptosis is a crucial final step in the removal of the MES. Indeed, using our novel sagittal imaging methods, we observed an abundance of apoptosis and apoptotic cell extrusion in MES cells during their removal. Furthermore, live imaging of epithelial islands confirmed epithelial loss through cell death, consistent with cell death being the ultimate fate of a substantial proportion of MES cells, particularly in the posterior palate. Loss of the intrinsic apoptotic regulators BAX and BAK resulted in a complete loss of MES cell death and prevented cell extrusion, indicating that cell death in the MES occurs through the intrinsic apoptotic pathway and that the bulk of extruded cells are apoptotic. Surprisingly, however, complete loss of cell death and cell extrusion did not dramatically disrupt MES clearance or secondary palate fusion, which, as we describe herein, proceeds through a progressive series of epithelial cell movements. First, small breaks in the MES sheet enlarge through the rearrangement of surrounding epithelial cells, and epithelia coalesce into a web-like network of trails and islands. The trails migrate as epithelial collectives through the surrounding mesenchyme and are either incorporated into the oral and nasal surfaces or eliminated through apoptotic cell extrusion. Cell migratory behaviors are maintained in the absence of apoptosis and cell extrusion and collective epithelial cell migration overcomes the loss of apoptosis to carry out MES cell removal (Fig. 9).

Previous attempts to observe cell behaviors during MES removal included static studies using *in vitro* culture of unpaired palatal shelves, which led to the conclusion that MES clearance does not depend on contact from the opposite palatal shelf (Charoenchaikorn et al., 2009; Takigawa and Shiota, 2004; Yamamoto et al., 2020). However, the patterns of epithelial removal that were described using these protocols were unlike what we observed using our approaches, suggesting that the cellular behaviors underlying epithelial loss in those models may be impacted by cell culture conditions. Indeed, unpaired palatal shelf culture performed in media that included amniotic fluid did not result in loss of epithelium (Takigawa and Shiota, 2004), which is also consistent with the fact that there are many mouse mutants that exhibit cleft palate because of defects in palatal shelf outgrowth, thereby resulting in a loss of palatal shelf contact, but which retain the palatal epithelium (Bush and Jiang, 2012). Taken together, the evidence is consistent with MES cell apoptosis and migration requiring palatal shelf contact for their initiation (Carette and Ferguson, 1992), but the molecular cues initiating migration remain unknown.

It has been repeatedly demonstrated that apoptosis does not occur upon loss of TGFβ3 signaling, which converges on Smad-dependent and Smad-independent pathways to regulate palate fusion (Lane et al., 2015; Xu et al., 2008). Given that TGFβ signaling is necessary for MES clearance, but apoptosis is not, TGFβ3 must also regulate other cell behaviors in this process. Indeed, TGFβ3 signaling regulates the differentiation of the MES, including the downregulation of ΔNp63, which in turn regulates numerous cell adhesion genes (Richardson et al., 2017). Furthermore, the compound loss of TGFβ3 and p63 in *Tgfb3*^−/−^*; p63^+/−^* embryos results in rescue of secondary palate fusion, providing functional support for this epistatic relationship (Richardson et al., 2017). Therefore, we were surprised to observe an abundance of cells co-expressing ΔNp63 and the periderm marker, Krt6a, in migrating epithelial trails. However, the increase in expression of Krt6a that we observed within basal epithelial cells fits well with previous studies that demonstrate that, similar to Krt6a, Krt17 expression is also increased within the basal epithelium of the MES during fusion (Jin et al., 2014; Vaziri Sani et al., 2005). Therefore, we hypothesize that these cells may represent an intermediate differentiation state as cells migrate in streams toward the oral and nasal surfaces. Richardson et al. also used a transgenic mKrt17-GFP reporter to demonstrate that the Krt17-expressing periderm cell layer underwent movement during palate fusion (Richardson et al., 2017). Our results are consistent with this finding, and further suggest that basal epithelial cells expressing ΔNp63 also take on periderm-like gene expression during migration, indicating that MES migration is concomitant to a change in basal epithelial differentiation state. Krt6a expression is also upregulated at other sites of epithelial fusion, such as the eyelids and embryonic wounds, and its loss affects the migration of keratinocytes during wound healing (Mazzalupo and Coulombe, 2001; Wojcik et al., 2000). It remains to be seen whether the change in expression of peridermal keratins, such as Krt6a, is involved in differentiation to a migratory cell phenotype or whether it is reflective of transitioning from a basal cell type to a surface cell type that must provide epithelial barrier integrity. Deep characterization of spatial changes in gene expression during palatal fusion will help to illuminate how differentiation is coupled with MES migration.

Several previous studies provided evidence that cell migration occurs during secondary palate fusion, but the nature of the migration and whether it is required for fusion were not clear (Carette and Ferguson, 1992; Kim et al., 2015; Richardson et al., 2017). We demonstrated that MES cells do not migrate as a sheet, but instead exhibit fascinating and unique patterns of epithelial cell movement, prompting several fundamental questions:

(1) How are initial breaks in the MES established? This does not require apoptosis, but could instead involve mesenchymal cells pushing through the MES or in a cell sorting-like behavior driven by differences in cell adhesion or actomyosin contractility between epithelial and mesenchymal cells (Kindberg and Bush, 2019; Lough et al., 2017). Matrix metalloproteinases are induced by TGFβ3 in the MES and are required for secondary palate fusion (Blavier et al., 2001), suggesting a role in the initiation of MES breakage. How the positions of these breaks are determined and how local diminution of MES cell-cell adhesion is balanced with overall retention of adherens junctions, which appear to be required for normal collective epithelial migration, is still mysterious.

(2) How are MES collectives guided to the oral and nasal surfaces, and do they exhibit cell polarity at the individual, or collective level? We did not observe lamellipodia or other protrusions in MES cell trails, and there was no apparent leading edge to the epithelial trails, which were invariably connected to the oral and nasal surface epithelium through a network of trails from the earliest stages. This begs the question of whether and how epithelial collectives know in which direction to migrate? Although islands also did not display directed cellular protrusions, they tended to follow the same path of previous epithelial trails when close to the oral or nasal edge, suggesting that the extracellular matrix (ECM) may act to guide epithelial collectives to the surface.

(3) How are cell fate decisions coupled with cell migration behaviors? Based on the migration patterns we observed, MES cells contribute to the nasal palatal surface, which will become respiratory pseudostratified ciliate epithelium, as well as the oral epithelium, which differentiates into oral stratified squamous epithelium. Whether MES cells are specified to these ultimate fates at the time they are undergoing migration, or after they reach their destination, may help us to understand how cell migration and fate specification are co-regulated.

We observed an enrichment of contractile filamentous actin at the epithelial-mesenchymal interface, and actomyosin contractility driven by NMIIA within the epithelium was crucial for collective migration. Given the importance of actomyosin contractility in reinforcing cell-cell junctions (Vicente-Manzanares et al., 2009), it is not surprising that loss of NMIIA resulted in a dispersal of MES cells. Future detailed studies will be needed to deconvolve the specific functions of actomyosin contractility between providing force for the migration of epithelial trails and its requirement for maintenance of epithelial collectives. Dispersed MES cells did not undergo EMT and also did not clear from the palatal mesenchyme, indicating that collective migration involving cell-cell adhesion is crucial. This finding is particularly notable in light of findings that *MYH9* and genes encoding multiple cell-cell adhesion components have been identified to be involved in cleft palate formation in humans and mice (Birnbaum et al., 2009; Chiquet et al., 2009; Cox et al., 2018; Jia et al., 2010; Lough et al., 2017, 2020; Peng et al., 2016). In addition, strong actomyosin contractions induce cell blebbing by the detachment of the cortical actomyosin cytoskeleton from the plasma membrane (Charras and Paluch, 2008). During apoptosis, caspase cleavage of ROCKI results in hyperactivation of NMII and promotes actomyosin contractility, resulting in membrane blebbing, formation of apoptotic bodies and disruption of nuclear integrity (Coleman et al., 2001; Croft et al., 2005). Therefore, it is notable that loss of BAX and BAK did not result in the loss of epithelial cell blebbing during MES migration. Cell blebbing is also thought to be involved in various forms of cell motility, including migration through a three-dimensional ECM (Charras and Paluch, 2008). Given that it has been previously shown that NMII-generated actomyosin contractility results in apoptosis in human embryonic stem cell culture (Chen et al., 2010), our observation that NMIIA-deficient embryos exhibited reduced cleaved caspase 3 staining raises the possibility that induction of MES apoptosis is a consequence of the high actomyosin contractility that moves the epithelial trails.

Live imaging of F-actin suggests that pulsatile actomyosin contractility, which is anisotropically enriched at the epithelial-mesenchymal interface and parallel to the direction of epithelial streaming, drives a peristaltic-like movement of epithelial trails. To our knowledge, this form of epithelial collective migration appears distinct from others that have been described in development, such as in the *Drosophila melanogaster* ovary and zebrafish lateral line (Rørth, 2009). Collective migration of neural crest cells in *Xenopus* occurs through supracellular actomyosin contractility at the rear of a migratory cell group, which drives intercalation of rear cells and forward movement (Shellard et al., 2018). Although MES migration also appears to occur through the modality of supracellular migration in which the scale of cell behavior is best described at the collective level (Shellard and Mayor, 2019), the precise mechanical drivers are likely different from NCC migration for at least two reasons. First, the rear of MES trails is not enriched for F-actin and, second, it seems unlikely that such localized actomyosin contractility would provide the force needed to push long, thin epithelial trails through the mesenchyme. Closer similarities might be found with cancer cells, in which actomyosin contractility drives the movement of an epithelial collective with stable cell-cell contacts; or with cell streaming, in which actomyosin contractility acts independently in each cell of a collective to allow cell rearrangement while maintaining transient cell-cell contacts (Friedl et al., 2012). In the future, localized manipulation of cell-cell adhesion and actomyosin contractility in the MES will help to elucidate the detailed mechanical drivers of this unique form of collective epithelial migration.

## MATERIALS AND METHODS

### Mouse lines

All animal experiments were performed in accordance with the protocols of the University of California, San Francisco Institutional Animal Care and Use Committee. Mice were socially housed under a 12 h light-12 h dark cycle with food and water. K6aiCre mice were generated by the CRISPR/Cas9 technology as follows: single guide RNA (sgRNA) targets were designed using the algorithm described by Haeussler and Concordet (2016). Chemically modified sgRNA was synthesized (Synthego) and tested for activity. sgRNA was complexed with enhanced-specificity Cas9 (eSpCas9) protein obtained from MilliporeSigma. A DNA donor was synthesized (BioBasic) to introduce the codon-optimized and ‘improved’ iCre recombinase (Shimshek et al., 2002) and a termination codon with the bovine growth hormone polyadenylation sequence at the Krt6a initiator methionine site in exon 1. The following reagents were microinjected into pronuclei of fertilized eggs: 30 ng/μl sgRNA+50 ng/μl Cas9 protein+10 ng/μl circular donor. Fertilized eggs were obtained by mating (C57BL/6 × SJL)F1 female mice with (C57BL/6 × SJL)F1 male mice. Eight candidate founder mice were identified by PCR amplifying over the 5′ and 3′ homology arms. The PCR products were subcloned, and 20 individual clones (both 5′ and 3′-flanking regions) from each sample were sequenced. Four of the candidate founder lines showed correct targeting without any undesired mutations. Two independent founders were used to establish the K6aiCre mouse lines. They both transmitted to the germ line with high efficiency. *Myh9^lox/lox^* (MGI: 4838521) and *Myh10^lox/lox^* (MGI: 4443039) mice have been previously reported (Jacobelli et al., 2010; Ma et al., 2009) and were maintained in a 129/Sv and C57BL/6J mixed genetic background. The following mouse alleles were backcrossed to, and maintained on, a congenic C57BL/6J genetic background: *Crect* (MGI: 4887352) (Reid et al., 2011), *Bax^lox/lox^* (MGI: 99702) (Knudson et al., 1995), *Bak*^−/−^ (MGI: 1097161) (Lindsten et al., 2000), *Rosa26^mTmG/mTmG^* (MGI: 3716464) (Muzumdar et al., 2007) and *Rosa26^nTnG/nTnG^* (MGI: 5504463) (Prigge et al., 2013). For genotyping, tail biopsies were collected at postnatal day 10 and either sent to Transnetyx or lysed for in-house PCR. For experimental analyses, embryos were harvested at E14.75-17.5, and littermates were used as controls when necessary.

### Immunofluorescence

For cryosection immunofluorescence experiments, whole embryos were fixed in 4% paraformaldehyde (PFA) in PBS, dehydrated through a sucrose gradient, embedded in OCT, and frozen in a dry ice/ethanol bath. Blocks were cut to 12 µm sections using an HM550 (Thermo Fisher Scientific) or a CM1900 (Leica) cryostat. Sections were then blocked in 5% normal donkey serum (Jackson ImmunoResearch) and 0.1% Triton-X-100 in PBS prior to incubation in primary antibodies at 4°C overnight. They were then washed with PBS and incubated in secondary antibodies at room temperature for 2 h. Slides were washed with PBS and mounted with Aquamount solution (Lerner Laboratories) before imaging.

For whole-MES immunofluorescence experiments, embryo heads were fixed in 4% PFA in PBS, embedded in 5% low-melt agarose, and sectioned in room-temperature PBS to 350 µm slices using a CT1000S (Leica) vibratome. Sections were washed in PBS, dehydrated through a methanol gradient, bleached in 15% H_2_O_2_ in methanol, and rehydrated. Sections were then blocked in 5% normal donkey serum and 0.5% Triton-X-100 in PBS prior to incubation in primary antibodies at 37°C for 24 h, washed with PBS, and then incubated in secondary antibodies at 37°C overnight. Sections were washed in PBS and dehydrated in a methanol gradient. Finally, tissue sections were cleared through a benzyl alcohol: benzyl benzoate (BABB)-in-methanol gradient (Ahnfelt-Rønne et al., 2007) before imaging. Images were captured using a Zeiss Cell Observer spinning disk confocal microscope or Zeiss laser scanning microscope and analyzed using Zeiss Zen software, Imaris software (Bitplane) and/or ImageJ. The following primary antibodies and dye were used in this study: anti-rat E-cadherin (Invitrogen, 13-1900, 1:300), anti-rabbit cleaved caspase 3 (Cell Signaling, 9661, 1:300), anti-goat p63 (R&D, AF1916, 1:300), anti-chicken GFP (Abcam, ab4729, 1:1000) and anti-rabbit ΔNp63 (BioLegend, 619001, 1:300). TUNEL staining was performed using an In Situ Cell Death Detection Kit (Roche, 11684795910) on coronal cryosections of 12 µm thickness.

### RNAscope *in situ* hybridization

Whole embryos were fixed in 4% PFA in PBS, dehydrated through a sucrose gradient, embedded in OCT, and frozen in a dry ice/ethanol bath. Blocks were cut to 12 µm sections using an HM550 (Thermo Fisher Scientific) or a CM1900 (Leica) cryostat. *In situ* hybridization was performed on the 12 μm sections using a *Cre* probe (Advanced Cell Diagnostics, 312281-C3) and RNAscope Multiplex Fluorescent Reagent Kit v2 (Advanced Cell Diagnostics, 323100) according to the manufacturer's protocol, with the exceptions of excluding antigen retrieval and reducing protease treatment to 5 min. The slides were then used following the cryosection immunofluorescence protocol for co-expression analysis (see above).

### Live imaging

The confocal live-imaging approach was adapted from our previous work (Kim et al., 2015, 2017), but applied on fresh secondary palate sagittal thick sections. Embryo heads were dissected and the top of the head, calvaria primordia and lower jaw were removed in ice-cold PBS. The remaining tissue, including the maxillae, was embedded in 5% low-melt agarose. Blocks were sectioned in ice-cold DMEM/F12 media to 250 µm slices using a Leica CT1000S vibratome. Regions of interest were confirmed by visualization of the endogenous EGFP reporter on a spinning disk confocal microscope. Sections were laid flat in a 35 mm No. 1.0 uncoated glass bottom dish (MatTek) and embedded in a mixture of agarose and culture media [20% fetal bovine serum (FBS), 2 mM L-glutamine, 100 U/ml penicillin, 100 µg/ml streptomycin, 200 µg/ml L-ascorbic acid to Dulbecco's Modified Eagle Medium (DMEM)/F12 media (Gibco DMEM/F12 without Phenol Red)]. This mixture was made by adding 3.5% low-melting agarose immediately prior to embedding, to a final concentration of 0.6%. For experiments that visualized F-actin, SiR-Actin (Spirochrome, CY SC001, 1:5000) was added to culture media immediately prior to the addition of agarose. Sagittal live imaging of the MES was performed using a Zeiss Cell Observer spinning disk confocal microscope or a Zeiss LSM900 laser scanning confocal equipped with a 37°C incubation chamber. Time-lapse images were captured with 488 nm and 647 nm (for SiR-Actin only) laser excitation for approximately 24 h at 15-min intervals. In these experiments, investigators were blinded to genotype (if applicable) until after completion of imaging. Movies of 3D renderings were generated using Imaris software. Bleaching correction was used to adjust the brightness of movies by adding key frames under the animation mode. For live imaging presented in Fig. 5B and Movie 8, the gamma was adjusted to 1.5 in order to visualize low-fluorescence epithelial trails without saturating the surface epithelium.

### Volume generation

All Zen (Zeiss) image files were converted into Imaris software (Bitplane) format and subsequently processed using Imaris. We segmented the MES using the epithelial marker E-cadherin to define a 3D surface (‘surface’ tool automatic mode in Imaris), manually removing nonspecific signals. The volume of the MES was then automatically determined from this generated surface. Mesenchymal volumes were calculated by subtracting the above-described E-cadherin volume from the total volume of the region of interest along the anterior-posterior (A-P) axis. Regions were taken from 30 µm image stacks. For segmenting trails versus triangles, we divided the MES into four equal parts by using the ‘grid’ function in Imaris. We defined the dorsal and ventral quarters as nasal and oral epithelial triangles, whereas the middle two quarters were composed of trails and islands. The surface of each quarter was manually created by following the palate shape. A surface of the E-cadherin signal and subsequent volume was then automatically generated for each region, as described above.

### Quantification and statistical analysis

To quantify apoptosis and cell extrusion, cleaved caspase 3, E-cadherin double-positive cells were manually counted on palate sagittal sections immunostained for E-cadherin and cleaved caspase 3 and counterstained with DAPI, with regions of interest selected according to the A-P axis. We represent the percentage of MES cells undergoing apoptosis as the apoptotic index, which was calculated as a ratio of casp3^+^Ecad^+^ cells to Ecad^+^ cells. In mutant cases in which E-cadherin-positive cells could not be reliably counted, a volume of E-cadherin signal (as described above in ‘Volume generation’) was used instead for normalization. Statistical analysis was performed using GraphPad Prism 8. Unpaired Student's *t*-tests were used to determine statistical significance. All raw data are presented as a collection in Table S1.

### Cell tracking and analysis

Individual nuclear EGFP signal was manually followed in 3D (‘spot’ tool in Imaris) at each time point to create cell tracks. The tortuosity of a cell track was calculated by dividing the overall displacement of the cell (defined by the first and last point of the track; Figs 3D and 4D) by the total length of the track (Figs 3C and 4C). The Y position at the beginning of imaging was in reference to the axis origin at the top left corner of the image.

## Supplementary Material

Supplementary information

Reviewer comments
