# Supplementary material for: A unique form of collective epithelial migration is crucial for tissue fusion in the secondary palate and can overcome loss of epithelial apoptosis
Source: Development. 2022 May 26;149(10):dev200181. doi: 10.1242/dev.200181 (PMC9188751; doi:10.1242/dev.200181)
Supplement: Supplementary information [file develop-149-200181-s1.pdf]

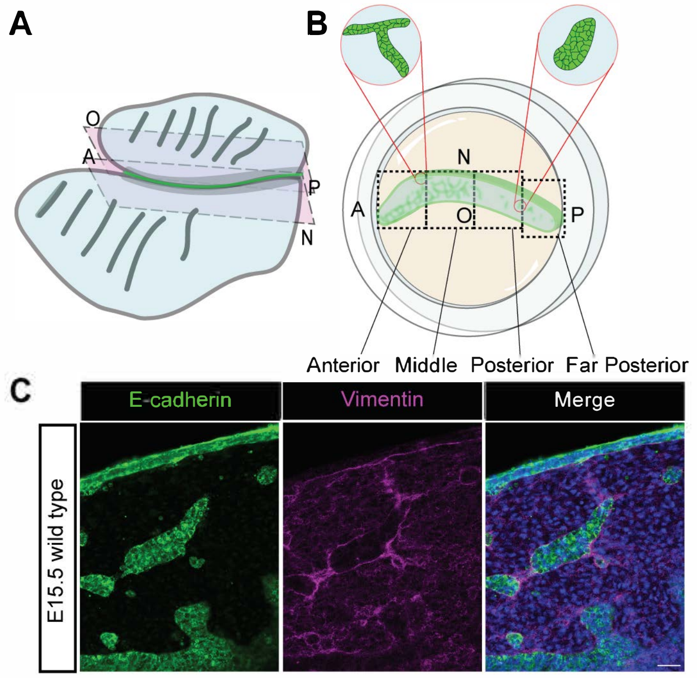

Fig. S1. Sagittal section imaging approach. (A) A schematic of an isolated mouse secondary palate, with the oral surface facing up, is trimmed using a vibratome to retain a thick section containing the MES region represented between the two dash-lined pink rectangles. (B) The thick section is placed in a glass bottom dish and mounted in a media-agarose mixture (orange). Views of an epithelial (green) trail and an island surrounded by mesenchyme (blue) are magnified in red circles. Black-dashed squares represent the different regions described throughout this paper. (C) 30-renderings of WT sagittal sections (n=2) immunostained for E-cadherin (green) and Vimentin (magenta) reveal epithelial trails mg rating through regions of palatal shelf mesenchyme.

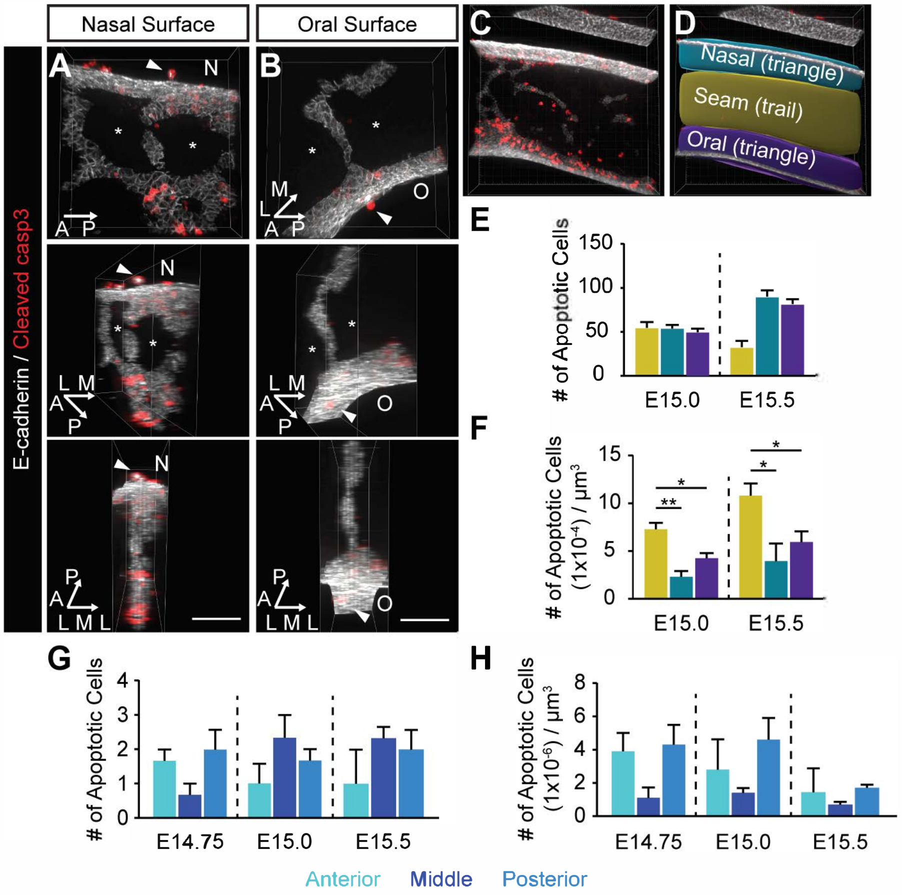

Fig. S2. Three-dimensional localization of apoptosis during palate fusion. (A,B) 3D-renderings of WT sagittal sections (n=3) immunostained for E-cadherin (white) and cleaved caspase-3 (red) are shown focusing at the region including the nasal surface (A) and oral surface (B). Sequential panels show the rendering turned at 45°and 90° on the nasal-oral axis. Arrowheads indicate extruded MES cells on nasal and oral surfaces. Asterisks indicate breaks in epithelium which are filled by unstained mesenchyme. N, nasal surface; O, oral surface; L, lateral; M, medial; A, anterior; P, posterior. Scale bar, 50µm. (C,D) A sample image of a E15.5 palate thick section immunostained for E-cadherin (white) and cleaved caspase-3 (red) (C) is segmented (D) to distinguish trail epithelium deep within the MES (yellow) and triangle epithelium at the nasal surface (turquoise) and oral surface (purple) for quantification in (E-F). To quantify within these regions, cleaved caspase-3 positive cells within surfaces of E-cadherin signal were counted at E15.0 and E15.5. (E,F) Histograms show the absolute number of apoptotic MES cells (E) in seam (yellow), nasal triangle (blue) and oral triangle (purple), as well as the number of apoptotic cells normalized to the volume of E-cadherin signal (F). Column height represents the mean from n=3. Error bars represent S.E.M, *, P<0.03; **, P<0.01. Statistical significance was determined by unpaired t-tests. (G-H) Quantification of apoptosis in mesenchyme during fusion (n=3 per stage). Histograms show the absolute number of apoptotic mesenchymal cells within a volume segmented by E-cadherin expression (see also Materials and Methods), at different positions of the secondary palate at E14.75, E15.0, and E15.5. (G) Column height represents the mean absolute number of apoptotic cells. (H) Column height represents mean apoptotic cell number normalized to mesenchyme volume. The mesenchyme volume is calculated as the volume of E-cadherin expression subtracted from the confocal stack volume within these regions.

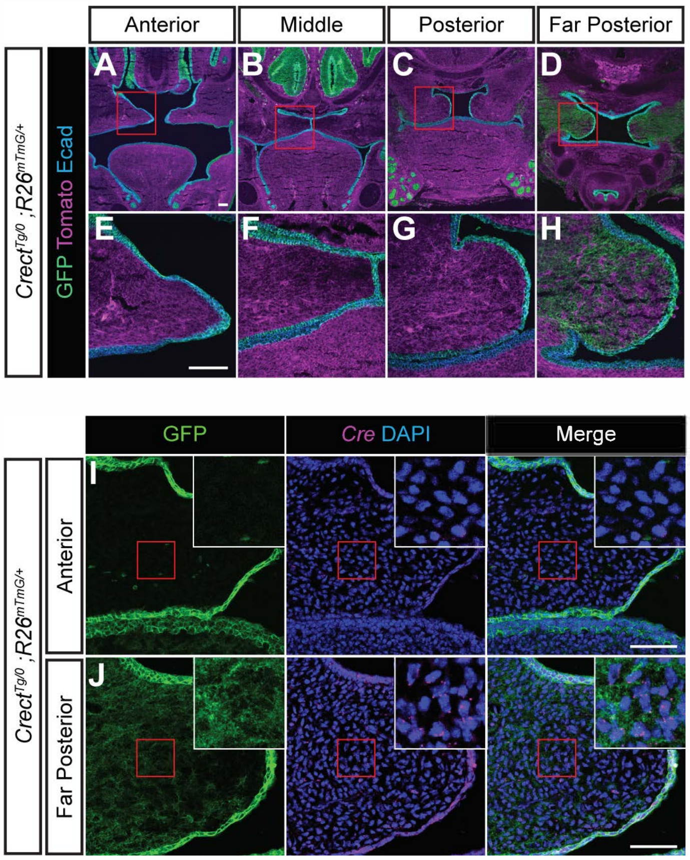

Fig. S3. Ectopic Crect activity limited to the far posterior palate. (A-D) Examination of Crect-mediated recombination in the secondary palate in *Crect*$^{Tg/0}$*; R26*$^{mTmG/+}$ secondary palates (n=9), (E-H) higher magnification views of regions designated by red boxes. Scale bars, 100µm. Near complete recombination (green) of the reporter can be observed in the epithelium (blue), with a lack of recombination observed in the mesenchyme (magenta) of the anterior (A,E), middle (B,F) and posterior (C,G) secondary palate. Extensive recombination of the reporter can be seen in the far posterior secondary palate mesenchyme (D,H). (I,J) Detection of *Cre* mRNA expression (magenta) by RNAScope *in-situ* hybridization relative to *Crect*$^{Tg/0}$*; R26*$^{mTmG/+}$ recombination in the anterior (I) and far posterior (J) palatal shelves of *Crect*$^{Tg/0}$*; R26*$^{mTmG/+}$ embryos. Inset is a high magnification view of the red square. Scale bars, 1 00µm.

Development • Supplementary information

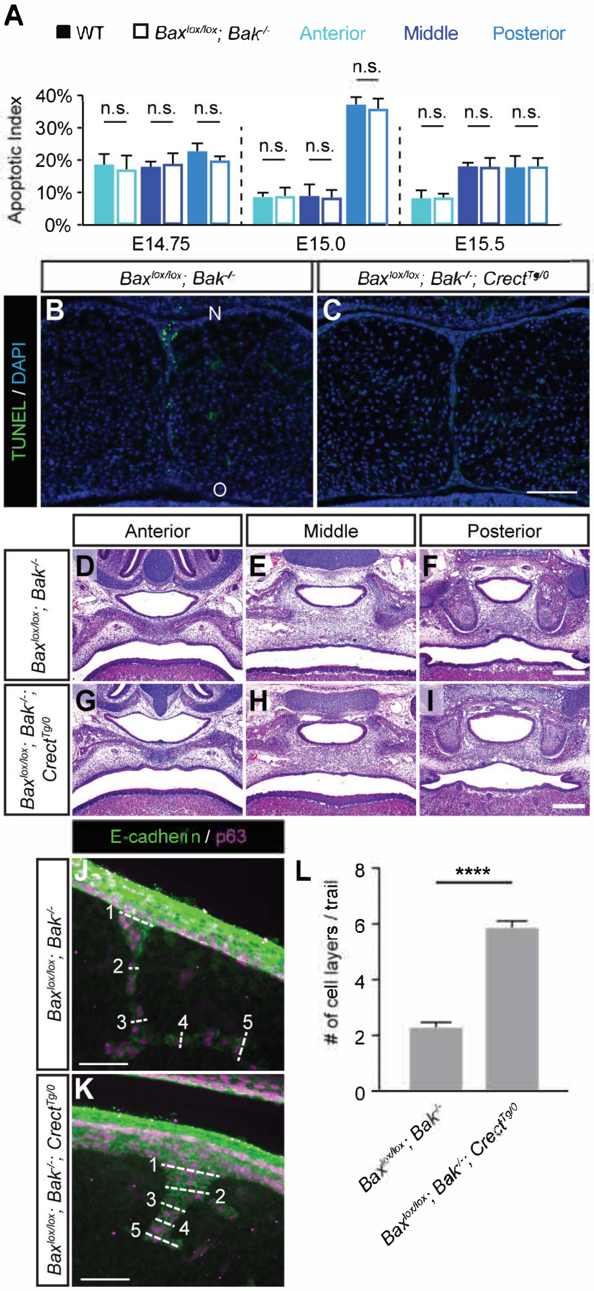

Fig. S4. Epithelial loss of Bax and Bak results in loss of epithelial cell death but does not prevent secondary palate fusion. (A) MES apoptosis is not significantly different between wild-type and *Bax*$^{lox/lox}$; *Bak* $^{-/-}$ embryos at E14.75, E15.0, and E15.5. Histograms show the apoptotic index, which is the ratio of the number of apoptotic MES cells to the total number of MES cells at each stage. Column height represents the means of the ratio for n=3 per stage. Error bars represent S.E.M; n.s., no significance. (B,C) TUNEL staining reveals an abundance of cell death in coronal sections of control *Bax*$^{lox/lox}$ *Bak*$^{-/-}$ secondary palatal shelves (B), and complete loss of cell death from the MES of *Bax*$^{lox/lox}$; *Bak*$^{-/-}$ *Crect*$^{Tg/0}$ embryos (C). Scale bar, 50 μm. (D-I) H&E-stained frontal sections of *Bax*$^{lox/lox}$; *Bak*$^{-/-}$ (n=3) and *Bax*$^{lox/lox}$; *Bak*$^{-/-}$; *Crect*$^{Tg/0}$ (n=3) secondary palates at E17.5 at anterior, middle, and posterior levels. Scale bar, 100 μm (J-L) The width of epithelial trails was measured in *Bax*$^{lox/lox}$; *Bak* $^{-/-}$ (n=3) and *Bax*$^{lox/lox}$; *Bak*$^{-/-}$; *Crect*$^{Tg/0}$ (n=3) E15.5 sagittal thick sections. (J,K) Trails were divided into five equal levels according to the total length of each trail, and the width at each level was measured by cell layers. Scale bar, 100μm. (L) Column height represents the mean width of trails. Error bars represent S.E.M; ****, P<0.0001. Statistical significance was determined by unpaired t tests.

Development • Supplementary information

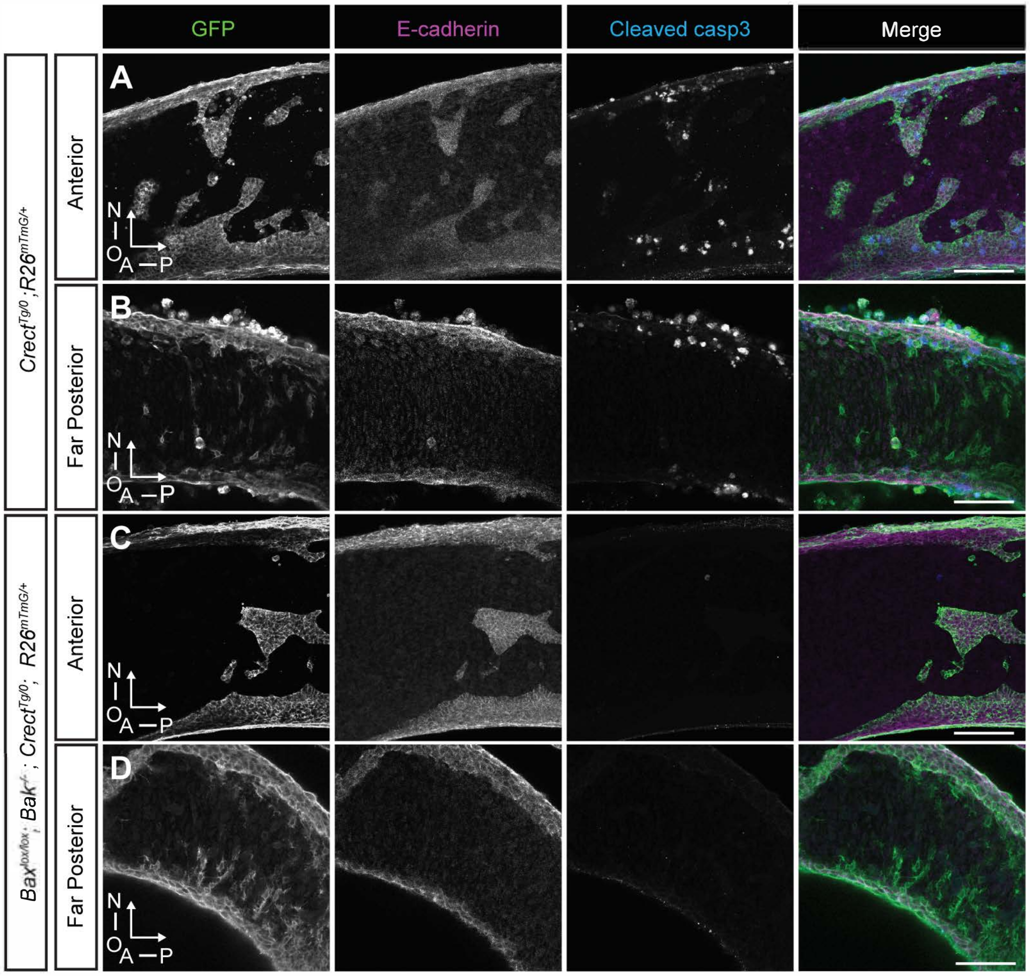

Fig. S5. EMT does not compensate for BAX/BAK loss in MES removal. (A-D) Epithelial lineage tracing and immunostaining of GFP (green) and E-cadherin (magenta) on sagittal thick sections reveals no GFP-expressing, E-cadherin negative cells in the anterior palate of control *Crect*$^{Tg/0}$; *R26*$^{mTmG/+}$ (A) or *Bax*$^{lox/lox}$; *Bak*$^{-/-}$; *Crect*$^{Tg/0}$; *R26*$^{mTmG/+}$ embryos (C). An abundance of GFP-expressing, E-cadherin negative cells were observed in the far posterior palate of control *Crect*$^{Tg/0}$; *R26*$^{mTmG/+}$ (C) and *Bax*$^{lox/lox}$; *Bak*$^{-/-}$; *Crect*$^{Tg/0}$; *R26*$^{mTmG/+}$ embryos (D). Scale Bar, 100μm. N, nasal surface; O, oral surface; A, anterior; P, posterior. N=5 for controls; n=6 for mutants.

Development • Supplementary information

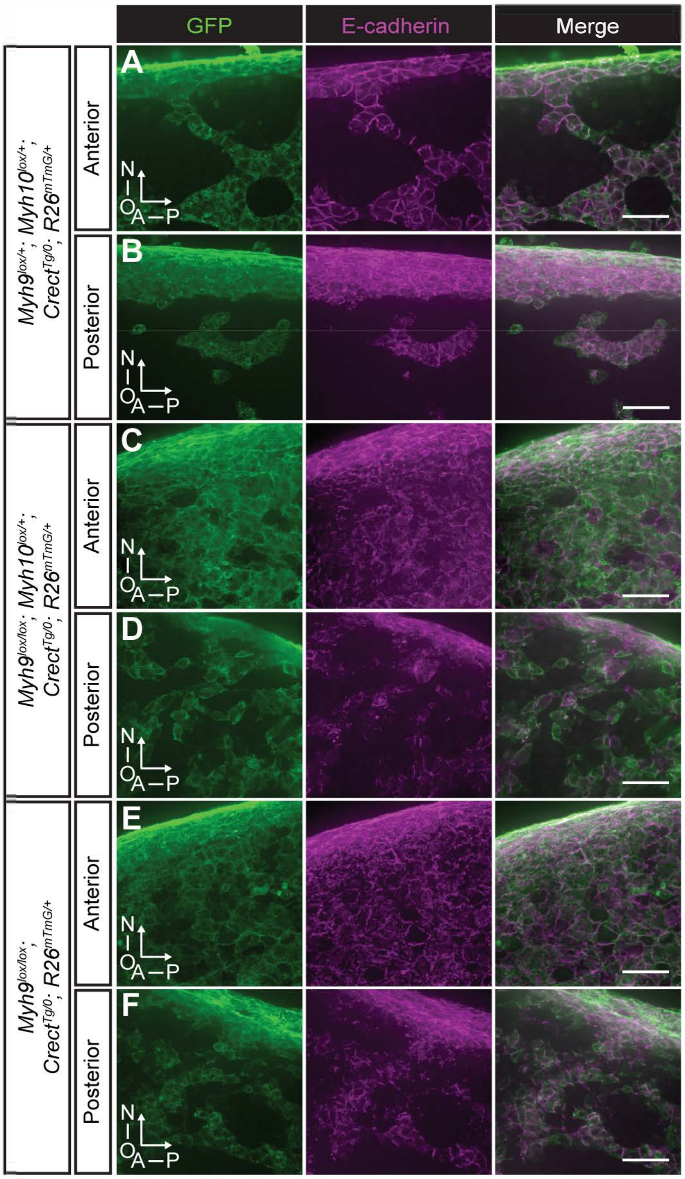

Fig. S6. NMIIA and not NMIIB is required for collective organization of the MES. 3D-rendered images of E15.5 control and NMII mutant palates visualizing *Crect*-mediated recombination (GFP in green) and MES cells, as labeled by E-cadherin in magenta. (A,B) In $Myh9^{lox/+}$; $Myh10^{lox/+}$; $Crect^{Tg/0}$; $R26^{mTmG/+}$ controls (n=5), trails and islands are detected in both anterior (A) and posterior (B) palate regions, with cell-cell junctions clearly revealed by E-cadherin expression. (C,D) In $Myh9^{lox/lox}$; $Myh10^{lox/+}$; $Crect^{Tg/0}$; $R26^{mTmG/+}$ (n=4) and (E, F) $Myh9^{lox/lox}$; $Crect^{Tg/0}$; $R26^{mTmG/+}$ (n=3) mutants, a large number of MES cells are broadly maintained at the seam in the anterior palate (C,E, respectively) and diffusely distributed in the posterior palate (D,F, respectively). Moreover, cellular distribution of E-cadherin is disrupted in mutant MES cells. Scale bars, 25μm.

Development • Supplementary information

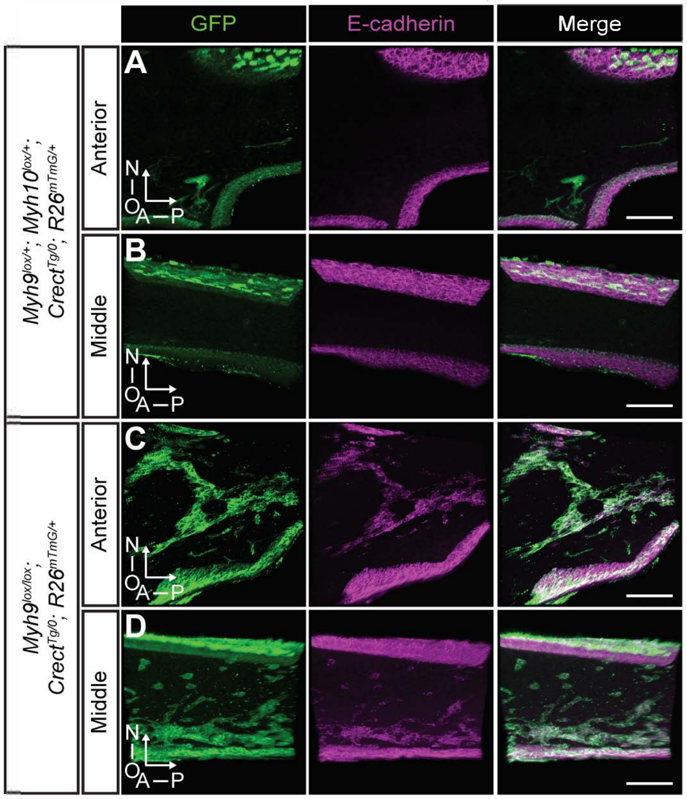

Fig. S7. Retention of epithelial inclusions in secondary palate upon loss of NMIIA. 3D-rendered images of secondary palate thick sagittal sections immunostained for GFP (green), to label *Crect*-mediated recombination, and E-cadherin (magenta), to label MES cells. (A,B) No MES cells are detected in $Myh9^{lox/+}$; $Myh10^{lox/+}$; $Crect^{Tg/0}$; $R26^{mTmG/+}$ control secondary palate at E17.5 (n=3). (C,D) Persistent MES cells are detected in the mutant $Myh9^{lox/lox}$; $Crect^{Tg/0}$; $R26^{mTmG/+}$ palate (n=3). A, anterior; P, posterior; N, nasal surface; O, oral surface. Scale bars, 100μm.

Table S1. Raw data for the values represented in the figures

Click here to download Table S1

Development • Supplementary information

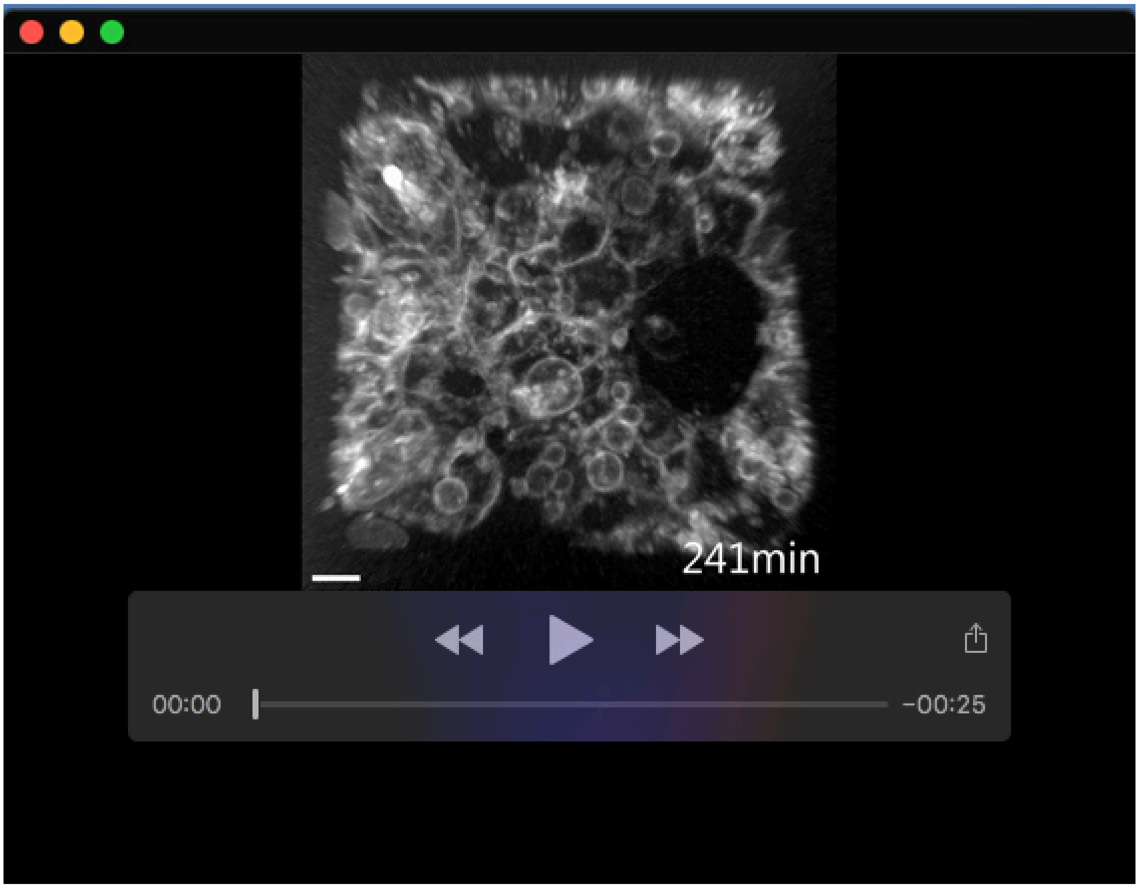

Movie 1. Live imaging of the initiation of MES breakdown during secondary palate fusion. Confocal live imaging of GFP expression in E14.75 *Crect*$^{Tg/0}$; *R26*$^{mTmG/+}$ secondary palate sagittal section shows MES breakage at an early stage of MES removal. Red arrowheads indicate membrane blebbing. Images were captured every 15 minutes for 20 hours. Scale bar, 15μm.

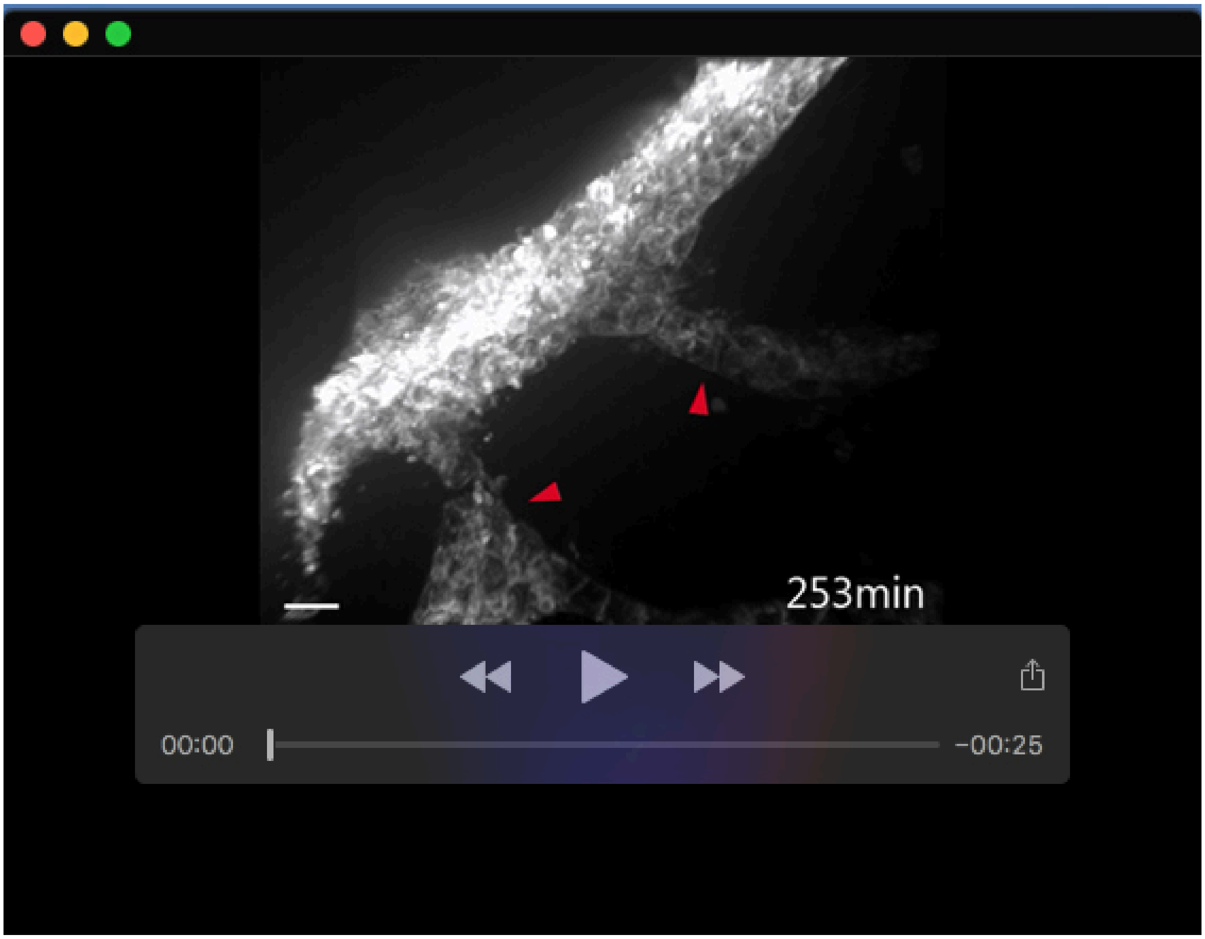

Movie 2. Organization and migration of MES epithelial cells in MES trails. Confocal live imaging of GFP expression in E15.5 *Crect*$^{Tg/0}$; *R26*$^{mTmG/+}$ secondary palate sagittal section shows MES trail movement as MES cells join the nasal epithelium and breakage of a trail to form an epithelial island. Arrowheads point to trails and broken ends of a trail. Images were captured every 15 minutes for 20 hours. Scale bar, 20µm.

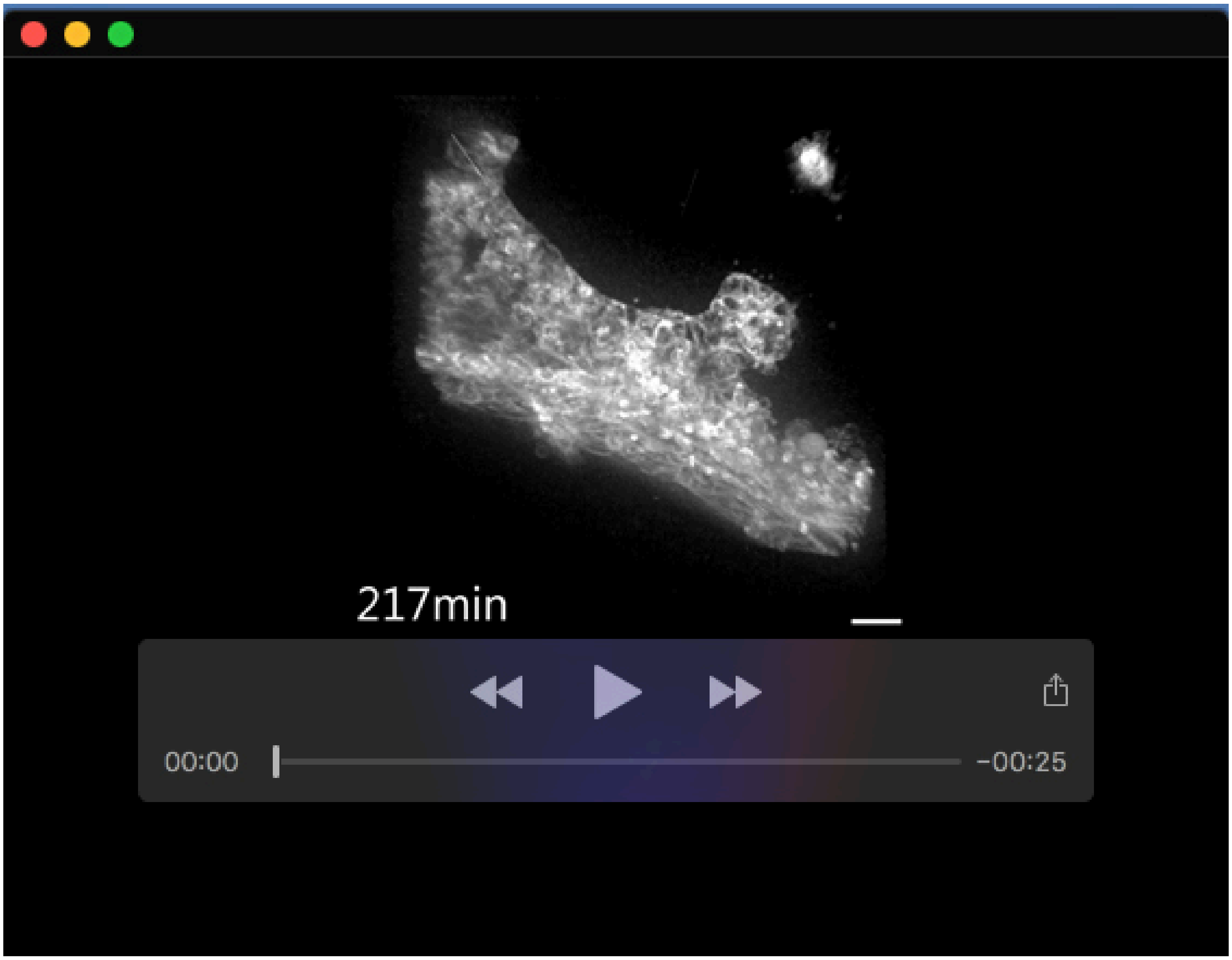

Movie 3. MES islands located near a surface epithelium removed by cell migration. Confocal live imaging of GFP expression in E15.5 *Crect*$^{Tg/0}$; *R26*$^{mTmG/+}$ secondary palate sagittal section shows an epithelial island coalescing into the nearby oral surface. Images were captured every 15 minutes for 20 hours. Scale bar, 20μm.

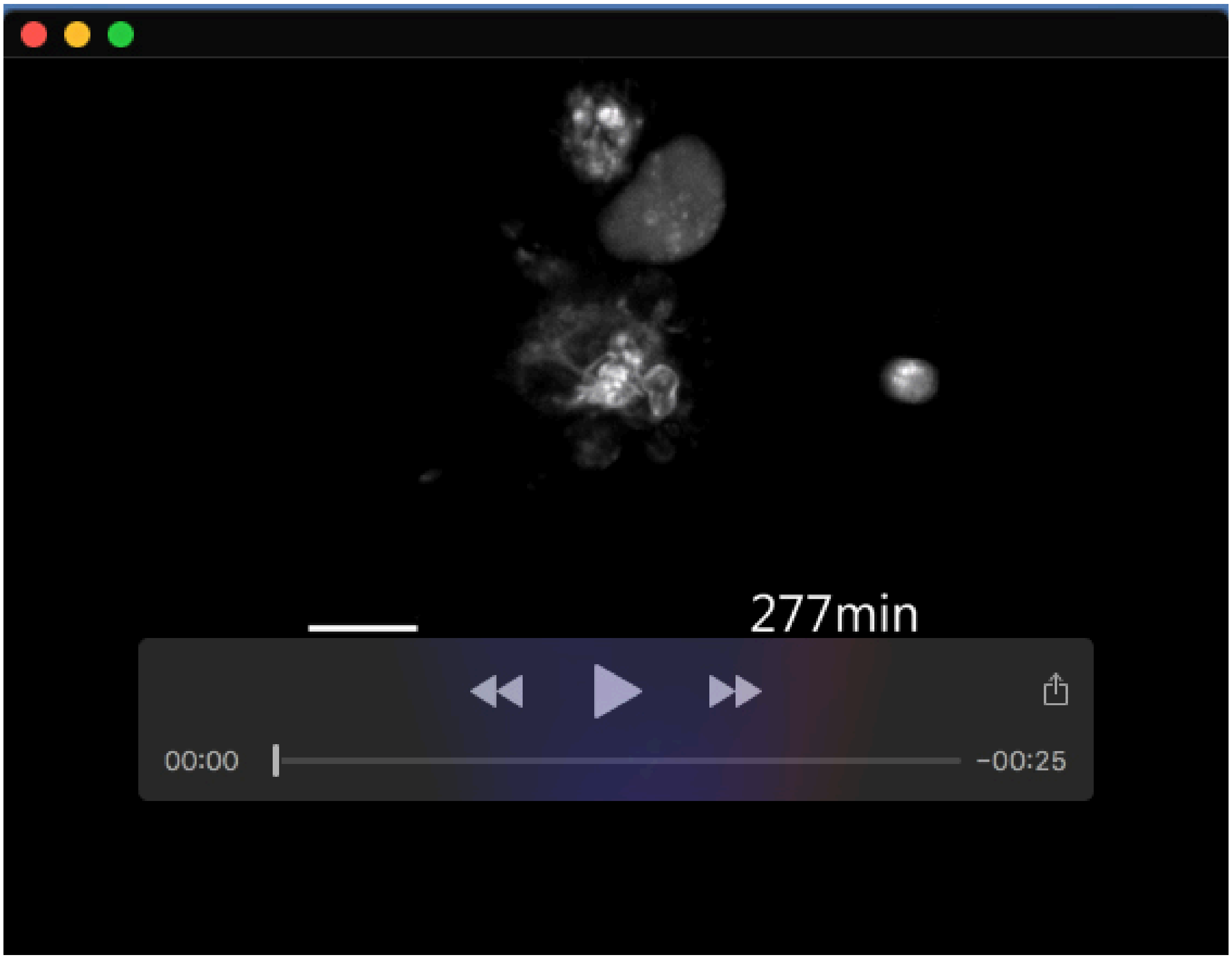

Movie 4. MES island located far from epithelial surfaces removed by apoptosis. Confocal live imaging of GFP expression in E15.5 *Crect*$^{Tg/0}$; *R26*$^{mTmG/+}$ secondary palate sagittal section shows an island that is distant from any epithelial surface exhibiting apoptotic appearance and progressive shrinkage. Arrowheads indicate characteristic blebbing behavior of apoptotic cells. Images were captured every 15 minutes for 20 hours. Scale bar, 15μm.

Development • Supplementary information

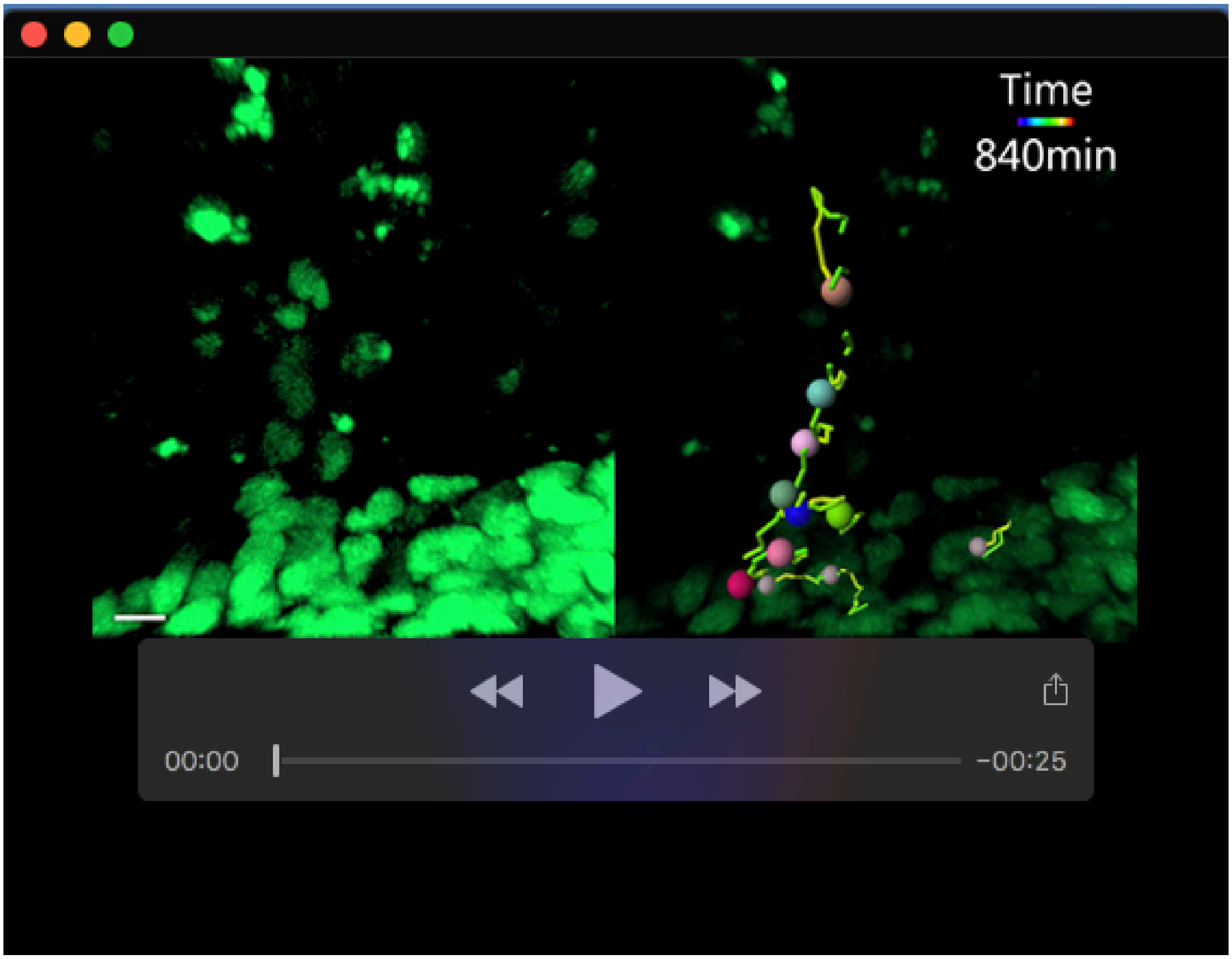

Movie 5. Epithelial trail cells collectively migrate in MES removal. Confocal live imaging of GFP expression in E15.5 *Crect*$^{Tg/0}$; *R26*$^{nTnG/+}$ secondary palate sagittal section shows a trail being incorporated into the oral epithelium (bottom of image). Bright GFP signal remaining in area adjacent to trail are likely apoptotic debris unconnected to trail cells. Animation on the right shows cell-tracks overlaid on the confocal imaging. Each tracked cell is represented by a colored sphere and respective time-coded tail. Images were captured every 15 minutes for 18 hours. Scale bar, 10 m.

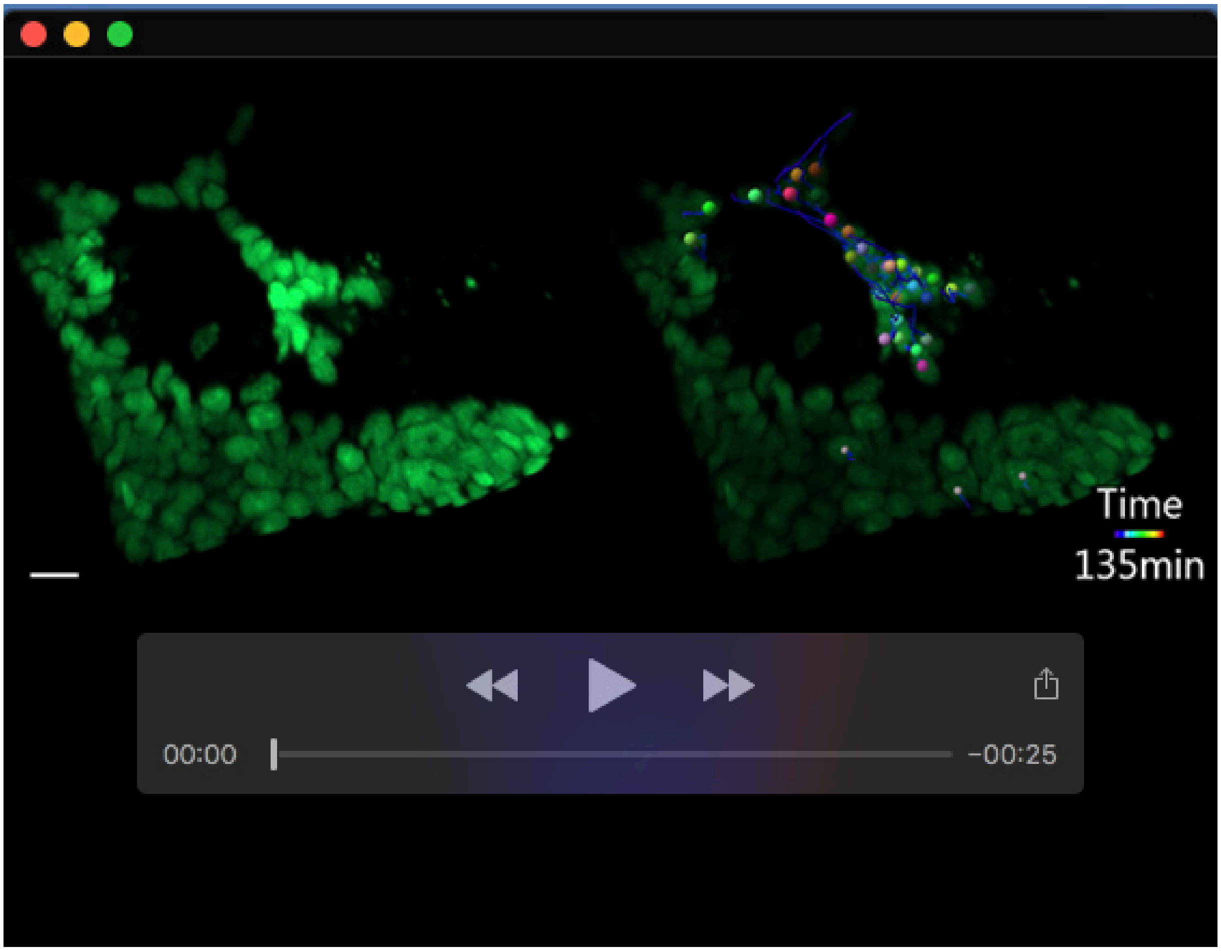

Movie 6. Epithelial island cells collectively migrate in MES removal. Confocal live imaging of GFP expression in E15.5 *Crect*$^{Tg/0}$; *R26*$^{nTnG/+}$ secondary palate sagittal section shows an island at the anterior palate being incorporated into the oral epithelium (bottom of image). Animation on the right shows cell-tracks overlaid on the confocal imaging. Each tracked cell is represented by a colored sphere and respective time-coded tail. Images were captured every 15 minutes for 10 hours. Scale bar, 15µm.

Development • Supplementary information

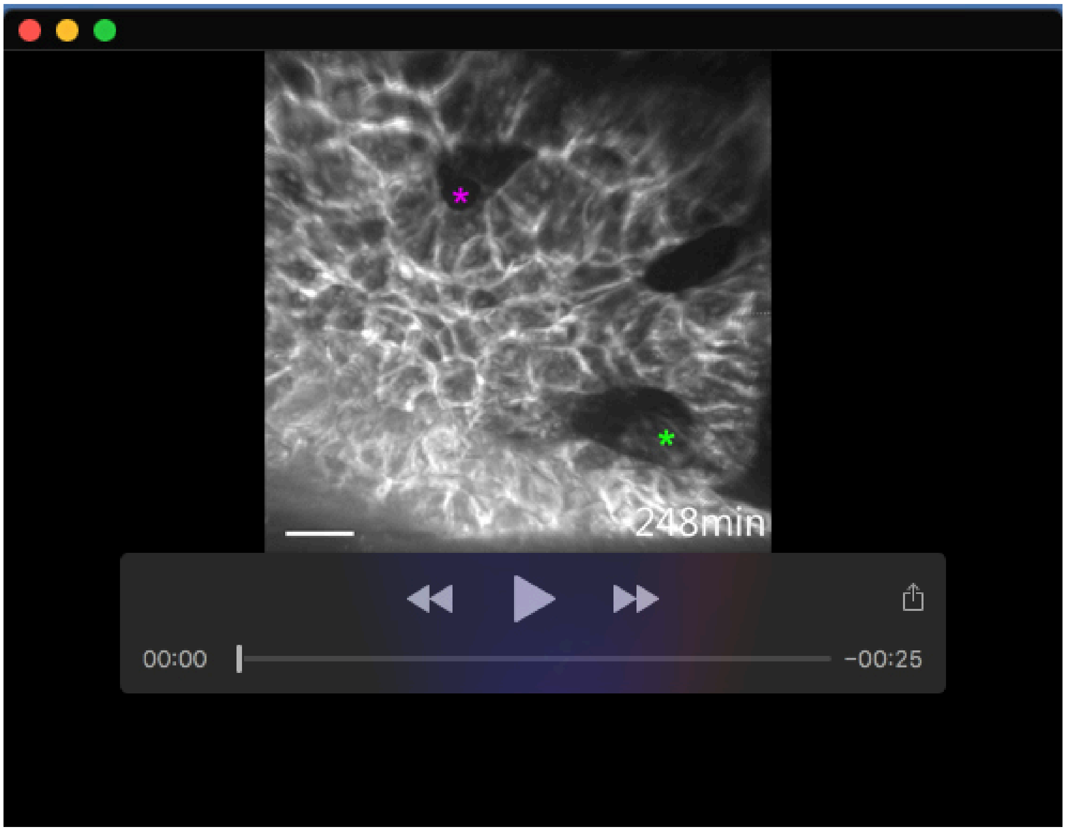

Movie 7. Loss of apoptosis does not disrupt MES breakage. Confocal live imaging of GFP expression in E14.75 *Bax*$^{lox/lox}$; *Bak*$^{-/-}$; *R26*$^{mTmG/+}$; *Crect*$^{Tg/0}$ secondary palate sagittal section shows the breakage of MES. Green asterisk indicates a separation of MES cells that continues to grow; magenta asterisk indicates a separation of MES cells that reduces and is eventually eliminated. Images were captured every 15 minutes for 20 hours. Scale bar, 15µm.

Development • Supplementary information

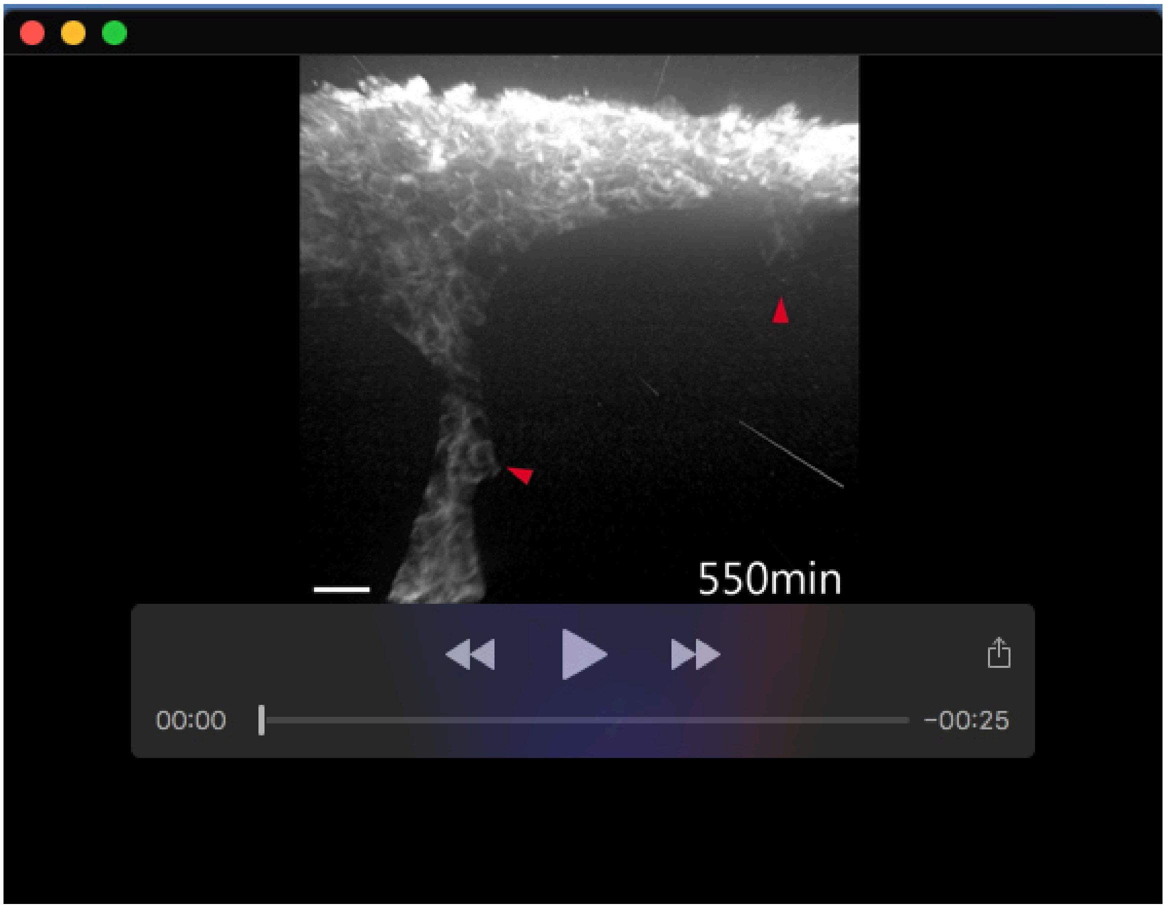

Movie 8. Loss of apoptosis does not disrupt collective epithelial migration. Confocal live imaging of GFP expression in E15.5 *Bax*$^{lox/lox}$; *Bak*$^{-/-}$; *R26*$^{mTmG/+}$; *Crect*$^{Tg/0}$ secondary palate sagittal section shows collective MES cell movement in trails. Arrowheads indicate trails in the movie. The gamma setting for this movie is adjusted to 1.5 to better visualize trails without saturating signal at the nasal epithelium. Images were captured every 15 minutes for 20 hours. Scale bar, 20µm.

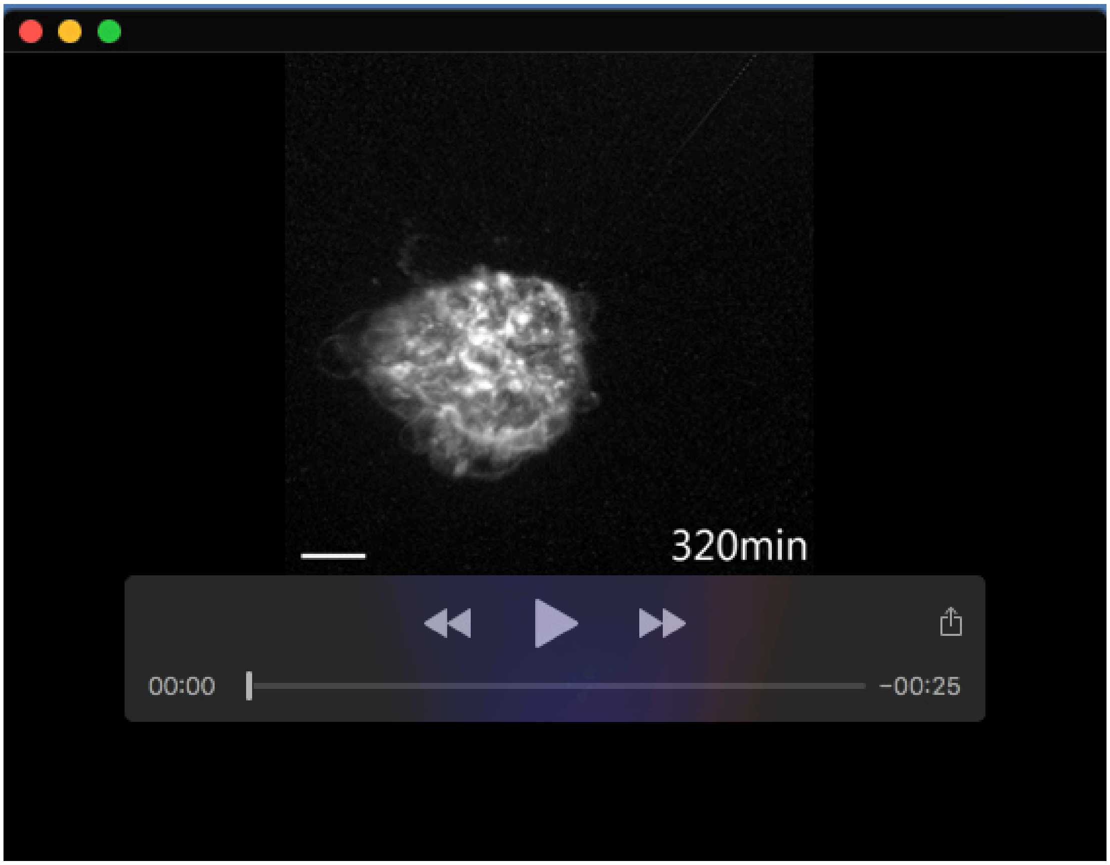

Movie 9. MES epithelial islands persist upon loss of apoptosis. Confocal live imaging of GFP expression in E15.5 *Bax*$^{lox/lox}$; *Bak*$^{-/-}$; *R26*$^{mTmG/+}$; *Crect*$^{Tg/0}$ secondary palate sagittal section shows the lack of MES cell removal of an island by apoptosis. Arrowheads indicate membrane blebbing that still occurs in mutants. Images were captured every 15 minutes for 20 hours. Scale bar, 15µm.

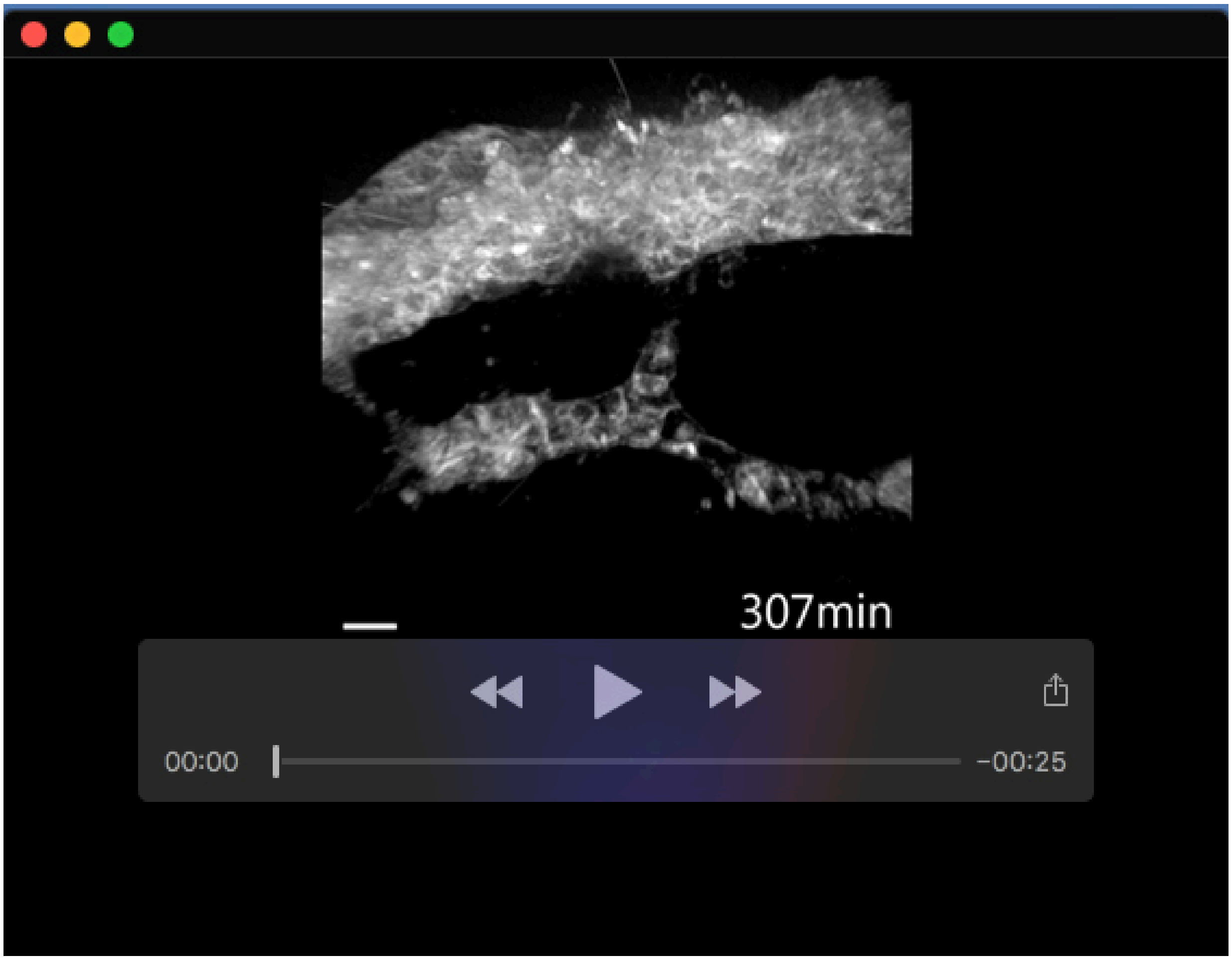

Movie 10. MES removal through collective epithelial migration. Confocal live imaging of GFP expression in control E15.5 *Myh9*$^{lox/+}$; *Myh10*$^{lox/+}$; *R26*$^{mTmG/+}$; *Crect*$^{Tg/0}$ secondary palate sagittal section shows normal MES cell movement in trails that incorporate into the nasal epithelium, along with breakage of the trail to form an epithelial island. Images were captured every 15 minutes for 20 hours. Scale bar, 15μm.

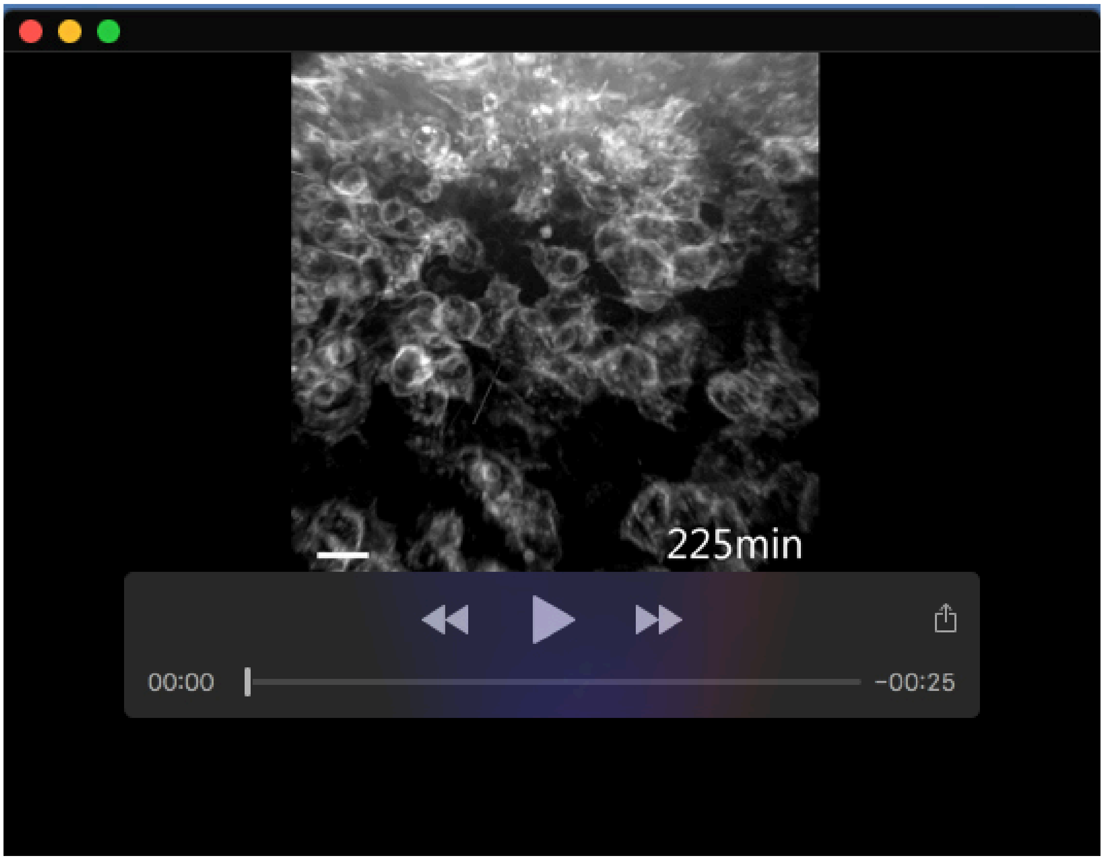

Movie 11. Collective organization and migration is disrupted upon loss of NMIIA. Confocal live imaging of GFP expressing cells in E15.5 *Myh9*$^{lox/lox}$; *R26*$^{mTmG/+}$; *Crect*$^{Tg/0}$ secondary palate sagittal section shows failure to form collective epithelial trails and islands and a lack of directional MES cell movement. Images were captured every 15 minutes for 20 hours. Scale bar, 15μm.

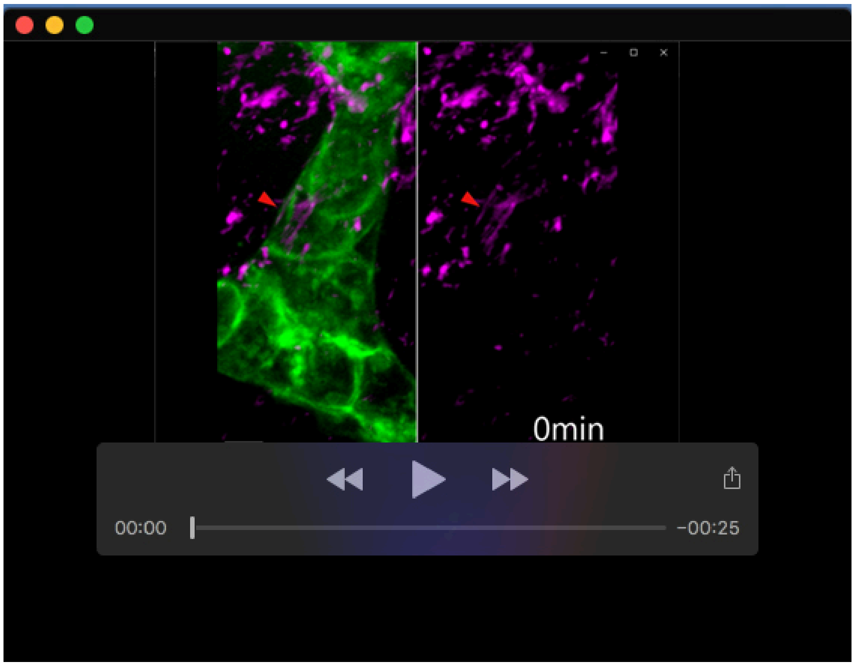

Movie 12. Anisotropic actin accumulation in epithelial trails drives collective epithelial movement. Secondary palate sagittal section of E15.5 control (*Myh9*$^{fl/+}$; *Myh10*$^{fl/+}$; *Crect*$^{Tg/0}$; *R26*$^{mTmG/+}$) embryos were stained with SiR-actin and then live imaged for GFP expressing MES cells (green) and F-actin (magenta). Arrowheads indicate anisotropic contracting F-actin during peristaltic MES cell movement. A contractile actin filament (arrowhead) can be seen adjacent to an MES cell of the trail. Scale bar, 5μm. Images were captured every 15 minutes. Frames shown in movie represent a 1-hour subset of a 20-hour time lapse shown in Figure 8A at a higher time-resolution.
