# Supplementary material for: A unique form of collective epithelial migration is crucial for tissue fusion in the secondary palate and can overcome loss of epithelial apoptosis
Source: Development. 2022 May 26;149(10):dev200181. doi: 10.1242/dev.200181 (PMC9188751; doi:10.1242/dev.200181)
Supplement: Reviewer comments [file develop-149-200181_review_history.pdf]

A unique form of collective epithelial migration is crucial for tissue fusion in the secondary palate and can overcome loss of epithelial apoptosis

Teng Teng, Camilla S. Teng, Vesa Kaartinen and Jeffrey O. Bush
DOI: 10.1242/dev.200181

Editor: Patrick Tam

Review timeline
Original submission: 7 September 2021
Editorial decision: 4 October 2021
First revision received: 16 March 2022
Accepted: 11 April 2022

Original submission

First decision letter

MS ID#: DEVELOP/2021/200181

MS TITLE: A unique form of collective epithelial migration is crucial for tissue fusion in the secondary palate and can overcome loss of epithelial apoptosis

AUTHORS: Teng Teng, Camilla Teng, Vesa Kaartinen, and Jeffrey O. Bush

I have now received all the referees' reports on the above manuscript, and have reached a decision. The referees' comments are appended below, or you can access them online: please go to BenchPress and click on the 'Manuscripts with Decisions' queue in the Author Area.

As you will see, the referees express considerable interest in your work, but have some significant criticisms and recommend revision of your manuscript before we can consider publication. If you are able to revise the manuscript along the lines suggested, which may involve further experiments, I will be happy receive a revised version of the manuscript. Your revised paper will be re-reviewed by one or more of the original referees, and acceptance of your manuscript will depend on your addressing satisfactorily the reviewers' major concerns. Please also note that Development will normally permit only one round of major revision.

We are aware that you may be experiencing disruption to the normal running of your lab that make experimental revisions challenging. If it would be helpful, we encourage you to contact us to discuss your revision in greater detail. Please send us a point-by-point response indicating where you are able to address concerns raised (either experimentally or by changes to the text) and where you will not be able to do so within the normal timeframe of a revision. We will then provide further guidance. Please also note that we are happy to extend revision timeframes as necessary.

Please attend to all of the reviewers' comments and ensure that you clearly highlight all changes made in the revised manuscript. Please avoid using 'Tracked changes' in Word files as these are lost in PDF conversion. I should be grateful if you would also provide a point-by-point response detailing how you have dealt with the points raised by the reviewers in the 'Response to Reviewers' box. If you do not agree with any of their criticisms or suggestions please explain clearly why this is so.

© 2022. Published by The Company of Biologists under the terms of the Creative Commons Attribution License (https://creativecommons.org/licenses/by/4.0/).

Reviewer 1

Advance summary and potential significance to field

The manuscript by Teng et al., titled “A unique form of collective epithelial migration is crucial for tissue fusion in the secondary palate and can overcome loss of epithelial apoptosis”, describes the mechanism of tissue fusion during secondary palate morphogenesis. The canonical view is that palate fusion requires apoptosis of the epithelial, although there is some contradictory evidence about this model. The authors genetically block epithelial apoptosis and find that tissue fusion and epithelial removal is largely unaffected. Instead, the authors find that epithelial removal is accomplished by collective migration of epithelial cells, and that cell extrusion is mainly occurring in apoptotic cells. Finally the authors proposes that actomyosin at the edge of the epithelial streams plays a key role in epithelial migration during epithelial removal at the palate.

This is an important novel contribution to our understanding of palate fusion, as it supports an alternative hypothesis different to the canonical model based on apoptosis to explain tissue fusion at the palate. The two main new conclusions in this manuscript are: 1) epithelial apoptosis is not essential for epithelial removal during palate fusion; 2) collective migration of epithelial cells contribute to epithelial removal. The data to support the first conclusion (apoptosis) are very sound and convincing, whereas the role of collective cell migration on epithelial removal requires some additional analysis (see specific comments).

Comments for the author

Specific comments:

1. My main problem with this manuscript is that it would benefit of a more quantitative approach to address some of the open questions
2. Although the authors claim that epithelial cells are removed by collectively migrating towards the surface epithelium, such directionally is not so evident in the movies provided. In each stream there are cells with low persistence, and some even move away from the surface epithelium. In addition the net movement of a whole stream or cluster seems that does not go in the direction of the surface epithelium (see for example the cell tracked in Fig 2E and corresponding movie: the bottom of the stream does not move upwards as expected)
3. How is the movement of epithelial clusters that are not directly connected with the surface epithelium? The example in Fig 6A suggest that between frames 10h and 20h the disconnected cluster become closer to the surface. The distance between each cell and the surface needs to be done to answer this kind of questions.
4. Considering the apparent low directionality of the moving cells, it is not clear how that could explain the almost complete removing of the epithelial with very little apoptosis. In my opinion extensive quantification of cell movement followed by a mathematical simulation could test the model put forward by the authors. In addition, a mathematical simulation could help to analyse the weight that different mechanisms (e.g., apoptosis, cell extrusion, cell migration) could have in epithelial fusion.
5. The authors show nicely that an actomyosin cable is formed at the edge of the epithelial clusters, and that global inhibition of myosin in epithelial cells impairs cell movement. Based on these experiments the authors conclude that the actomyosin cable is required for epithelial movement. However, this conclusion is premature, as the genetic inhibition of myosin performed in this manuscript could affect other actomyosin process different to the contraction of the actomyosin cable at the edge cluster. In order to test their hypothesis, the authors need to interfere specifically with the actomyosin cable at the edge cluster, which could be done by laser ablation or optogenetics.
6. Inhibition of myosin leads to single cell migration, suggesting that myosin is required to maintain cell-cell adhesion, as it has been previously published. In addition, these observations suggest that the reason why these single cells do not migrate correctly, is because they need to migrate collectively. This could be directly tested by blocking cell-cell junction in the epithelial cells, followed by analysis of migration.
7. The authors argue that although apoptosis plays a role in epithelial remotion, apoptosis inhibition does not affect removing of the epithelial at large. To sustain this apparently contradictory statement, the authors need to quantitatively compare epithelial behaviour with and

© 2022. Published by The Company of Biologists under the terms of the Creative Commons Attribution License (https://creativecommons.org/licenses/by/4.0/).

without apoptosis. A possible explanation is that in absence of apoptosis alternative mechanism, such as migration, are stimulated to compensate for the lack of apoptosis removal.
This need to be properly analysed.

Reviewer 2

Advance summary and potential significance to field

As is the custom for the Bush lab, this study makes use of elegant mouse genetics and cutting-edge imaging techniques to explore the dynamics of palate closure here with the focus of resolving the conflicting reports on the role of apoptosis in medial epithelial seam dissolution during palatal shelf fusion. This is a very important question, as there have been a number of studies implicating the cell death machinery as being critical (or not) for palate fusion. Ostensibly, with the tools these authors have on hand, they have the potential to finally resolve the long-standing controversy of how important apoptosis is for palatal fusion, and whether it functions primarily in the epithelial or mesenchymal compartment.
However, in some regards they fall short of this goal, and many of my specific comments are aimed at using tools in hand to more definitively address this issue.
In addition to looking at regulators of apoptosis such as Bax and Bak—which the authors conclude are dispensable for palate closure, but do play a minor role in midline epithelial seam (MES) removal—they utilize their novel tools to revisit the role of non-muscle myosins IIA/B (Myh9 and Myh10) in palatogenesis. They use live imaging in their new sagittal thick-section preparations to show that epithelial cells migrate collectively, and develop a new periderm Cre driver (Krt6a-iCre) which suggests that basal epithelial cells—in addition to periderm—may participate in these migrating cell "trails." Overall, the authors conclude that collective migration requires myosins, and that this coordinated migration is more important for MES resolution than apoptosis (though it appears that neither on its own is sufficient to cause CP when using their Crect-Cre driver). The imaging and movies are beautiful, the data are clearly presented and of high-quality, and the Discussion is thoughtful and thorough. Quantifications are provided where important, and n values and statistical tests appear to be appropriate. This is a study that should be of interest to readers of Development and is in the upper echelon of papers I've read and reviewed for this journal. Even if the authors cannot unambiguously resolve the issue of the relative contributions of apoptosis in epithelial vs. mesenchymal populations, I still support publication of this high-quality study in Development.

To provide a bit more background on the role of apoptosis in palatogenesis, I will briefly summarize the past literature here, as I understand it. More than 20 years ago, the apoptotic regulator Apaf1 was shown to be essential for palate closure as germline mutants display palatal shelves that meet at the midline but do not fuse (Cecconi, 1998; Honarpour, 2000). However, conditional epithelial-specific deletion of Apaf1 using the Krt14-Cre driver does not result in CP (Jing & Dong 2006), despite a reduction in TUNEL+ cells. A caveat of these findings is that this particular Krt14-Cre driver (from Elaine Fuchs) may not be fully functional in palatal epithelium (see review by Lough et al, 2017). Even though the authors show Cre expression and function by in situ and Rosa-lacZ reporter, they fail to show whether Apaf1 is functionally deleted at the protein level during palate closure. Thus, this negative data cannot, in my opinion, formally rule out a requirement for Apaf1 in the epithelium. Additionally, a recent report of global KO of the apoptotic effectors Bax and Bak (the same genes studied here) showed ~30% penetrance of CP at E18.5, with a slightly higher proportion (45%) when Bok is also deleted. The present study is the first to conditionally ablate Bax and Bak in the epithelium using the Crect-Cre driver, and they report a lack of CP.
Thus, collectively these studies suggest that apoptosis is necessary for palate closure, but that it may be less important in the epithelial compartment.
Unfortunately, to my knowledge, no study has generated mesenchymal-specific KOs of apoptotic regulators, so the relative importance of this pathway in the palatal mesenchyme has not been directly tested. The lack of a strong phenotype via Crect-Cre-mediated deletion of Bax and Bak, is in my opinion still compounded by several issues: 1) the latest age examined is E15.5, so it cannot be ruled out that palate closure is delayed, or CP may ultimately develop, 2) Crect-Cre appears to be epithelial-specific in anterior palatal regions, but more broadly expressed in posterior palate.

© 2022. Published by The Company of Biologists under the terms of the Creative Commons Attribution License (https://creativecommons.org/licenses/by/4.0/).

Comments for the author

Specific comments:
1. Fig. 1A-C: It would be nice to see a mesenchymal cell stain (or at least DAPI) in addition to epithelial Ecad. It is difficult to discern whether the formation of these epithelial trails accompanies general changes in local cell density or whether the mesenchymal microenvironment appears fairly unchanged. The use of the term “small epithelial holes appeared” (p. 5) implies these might be acellular areas, so it should be clarified if this is not the case.
2. What is the proportion of mesenchymal cells that are cC3+? While it appears that most are epithelial, a greater number in the posterior palate appear to be mesenchymal from Fig. 1C. This becomes important later when the authors note that the Crect-Cre driver is active in posterior palatal mesenchyme (see related comments 4, 9).
3. How much apoptosis is occurring within “trails” inside the MES as compared to epithelial “triangles” at the nasal and oral surfaces?
4. The Crect-Cre mice used in this study (Reid et al, 2011) were previously characterized for their ability to act in craniofacial surface ectoderm beginning around ~E10.5. However, it is not clear when/where it is acting in oral epithelium during palate closure. From p.7 Results: “we did not observe any GFP reporter positive, E-cadherin negative cells throughout most of the palate,” this suggests it is epithelial-specific (at least anteriorly). However, this could be conclusively demonstrated using their mTmG reporter (Fig. S2) by showing the Tomato channel (where Cre is inactive) in addition to the GFP channel (where Cre is active). Similarly, in the Crect-Cre; R26nT/nG experiments (Fig 2E,F), it would be very nice to be able to visualize the red (mesenchymal) nuclei as well as the green (epithelial) ones, which are shown.
5. It is curious that in Crect-Cre; Baxfl/fl; Bak-/- mice, apoptosis by both TUNEL and cC3 appears to be completely absent, even in the mesenchyme, although this is only quantified for Ecad+ cells 6. Bak-/- KO mice are used as the control group for most of the experiments. Is there any difference in the degree of apoptosis between these and WT mice?
7. Palate closure in Crect-Cre Baxfl/fl; Bak-/- mutants is only investigated at E15.5, when it is concluded that PS fuse normally and “MES clearance complete[s] successfully,” but display abnormal migration. Previous studies of cell death mutants show that PS meet but do not fuse at E15.5, and global Bax; Bak KOs show a high penetrance (30%) of CP by E18.5. MES clearance is later ages should be investigated to determine is clefting occurs or may be delayed in Crect cKOs.
8. Results, p. 8 “we generated a Krt6a iCre knock-in mouse line for tracking the migration of peridermal cells.” As far as I can tell, the method for generating this mouse is not described in the Methods section and no reference is provided.
The term “iCre” is also not defined here and is ambiguous. Some groups use this term to refer to “inducible” (e.g. tamoxifen regulable CreER), while it can also mean “improved” (e.g., codon-optimized). I assume it is the latter here as no mention is made of tamoxifen regimens, but this should be clarified in the text.
9. According to the text, only “mid-anterior MES” was imaged for the studies in Figs. 2/3. Given the unexpected finding that Crect-Cre is active in the posterior palatal mesenchyme, the authors should make use of this fortuitous finding to compare MES dissolution in the anterior and posterior palate region. Presumably all apoptosis (mesenchymal and epithelial) will be blocked in the posterior region of Crect-Cre; Baxfl/fl; Bak-/- mice while only epithelial apoptosis will be blocked in the anterior region.
10. Results, p. 9: “p63-expressing basal epithelial cells undergo migration to the oral and nasal surface concomitant with the basal epithelial initiation of expression of periderm marker Krt6a.” In my opinion, since I think the Krt6-iCre line used here is non-inducible, one must consider an alternative explanation which is that migrating cells expressed Krt6a before initiating migration, rather than turning it on during the migration process. In fact, from the merged images in Fig. 4, it would appear that quite a few cells express either Krt6a or p63 heterogenously, as there are very few white (magenta + green) cells even in the trails. Again, here, I would love to see the red nuclei (Krt6a-iCre-negative) as well as the green. In any case, since there is a pretty strong precedent from the Dixon lab that periderm cells can migrate during MES dissolution, perhaps the authors could be more cautious in their conclusion and could perhaps preface this statement with “in addition to periderm, p63-expressing epithelial cells undergo migration…” Live imaging of the Krt6a-iCre; R26nTnG line could also unambiguously resolve whether Krt6a is turning on as cells migrate, though I concede this is a lot of work to ask for.
11. I don’t believe it is explicitly stated in the text whether CP is ever observed in the Crect-Cre; Myh9fl/fl; Myh10fl/+ mice, though it is written that “epithelial inclusions persist” even at E17.5,

© 2022. Published by The Company of Biologists under the terms of the Creative Commons Attribution License (https://creativecommons.org/licenses/by/4.0/).

which is reminiscent of the persistent MES phenotype previously described in Krt14-Cre; Myh9fl/fl and Tgfb3-Cre; Myh9fl/fl mice (Kim et al, 2015) by this group. I believe that this earlier paper only reported CP in Tgfb3-Cre; Myh9fl/fl; Myh10fl/fl double mutants, so what is not clear to me is whether the apparent lack of CP here is due to the residual copy of Myh10 or the use of the Crect-Cre driver. I am not sure why the authors excluded data on Crect-Cre; Myh9fl/fl; Myh10fl/fl double KO mice when they went to the trouble to examine Crect-Cre; Myh9fl/fl; Myh10fl/+ and Crect-Cre; Myh9fl/+; Myh10fl/fl genotypes.

12. There appears to be a discrepancy in the apoptotic behavior between anterior and posterior palatal regions reported in Figs. 1 and 5. In Fig. 5, there is a gradient of apoptosis that is higher in anterior and middle palate compared to posterior, in both WT controls (Myh9fl/fl; Myh10fl/fl) and Myh9/10 mutants.

However, in Fig. 1D-E, there appear to be greater numbers of cC3+ cells in the posterior compared to anterior region at every age examined (E14.75-E15.5). Both examine cC3+ cells in different palate regions at E15.5 using the sagittal thick-sectioning technique, so I am not sure what can explain this dramatic difference between experiments.

13. When cC3 is shown in blue (e.g. Fig S2, Fig. 5), it is extremely difficult to see. I suggest showing single channel images in gray, or another color.

Reviewer 3

Advance summary and potential significance to field

In this well drafted manuscript with high level imaging data from mouse model, Teng et al. describe in detail how epithelial cells of the MES migrate out of the seam during secondary palate fusion. Further, they characterize the role of apoptosis in this process. These analyses were possible due to their application of a live cell imaging technique.

Major Comments:

1. Introduction immediately starts on discussing the secondary palate. To make the paper accessible to readers (scientists and clinicians), it is important to briefly make the following points. 1) the palate is made up of primary and secondary palate, there are bony and muscular portions, 2) functions of the palate and clinical relevance, 3) genetic pathways underlying primary palate and secondary formation are likely distinct with some overlaps and 4) mouse models focus on secondary palate. This context is important from the outset to frame the specific question being investigated here. I think most craniofacial biologists don't appreciate the significant differences of primary vs. secondary palate development. The Discussion should then take the data presented here and place it in the context of total palate development, how lessons from this epithelial collective migration apply, or not, to primary palate development, which is more clinically prevalent (CLP is observed more than CPO).
2. There is no description of how the Krt6a iCre mouse was generated or validated. This is important since there is no consensus in the field on what marker truly labels the periderm and each mouse group seems to choose their favorite or what they have breeding. Consider citing Trevor Williams and characterization of the Crect line.
3. What is the state of the palate in the Myh9/Myh10; Crect compound mutants? Are these mice viable?
4. In Figure 1 and Figure 5 it is unclear how cell numbers were determined (number of apoptotic MES cells to total number of MES cells). How were cell boundaries determined?
5. The cell imaging results are not matched with molecular data, which is a missed opportunity. With emerging molecular detail in mouse and zebrafish models, scRNAseq studies, genetic mutant analysis of cleft models, it would be more informative to connect some of the cell behavior to the genes that regulate the process. The study visualized p63 and cadherin, both genes have been implicated in molecular regulation of palate development in model organisms and human CLP cohorts, but molecular or genetic analysis of this cell migration process are lacking here.
6. There is no comment on the morphology of the lip and primary palate in the Crect BAX model here. It would be surprising if broad epithelial deletion of BAX in periderm does not cause lip/facial or primary palate phenotypes. Please show data.
7. Crect expression is much broader than periderm and is variable among litters, or even pups within litters – how was this variability and lack or periderm specificity accounted for in the experiments? Recommend confirming results with another epithelial Cre driver?

© 2022. Published by The Company of Biologists under the terms of the Creative Commons Attribution License (https://creativecommons.org/licenses/by/4.0/).

In total, nice study, very focused on secondary palate, but the study should be placed in context of palatogenesis and could be more impactful if there was molecular detail to go along with the cell migration descriptions.

Minor Comments:
1. In figure 2 and figure 3, do the time point labels apply to the other non-labeled images in the panel? For example, in 2C are those also 0, 5, 10, 15 and 20h?

Comments for the author

In this well drafted manuscript with high level imaging data from mouse model, Teng et al. describe in detail how epithelial cells of the MES migrate out of the seam during secondary palate fusion. Further, they characterize the role of apoptosis in this process. These analyses were possible due to their application of a live cell imaging technique.

Major Comments:

1. Introduction immediately starts on discussing the secondary palate. To make the paper accessible to readers (scientists and clinicians), it is important to briefly make the following points. 1) the palate is made up of primary and secondary palate, there are bony and muscular portions, 2) functions of the palate and clinical relevance, 3) genetic pathways underlying primary palate and secondary formation are likely distinct with some overlaps and 4) mouse models focus on secondary palate. This context is important from the outset to frame the specific question being investigated here. I think most craniofacial biologists don't appreciate the significant differences of primary vs. secondary palate development. The Discussion should then take the data presented here and place it in the context of total palate development, how lessons from this epithelial collective migration apply, or not, to primary palate development, which is more clinically prevalent (CLP is observed more than CPO).
2. There is no description of how the Krt6a iCre mouse was generated or validated. This is important since there is no consensus in the field on what marker truly labels the periderm and each mouse group seems to choose their favorite or what they have breeding. Consider citing Trevor Williams and characterization of the Crect line.
3. What is the state of the palate in the Myh9/Myh10; Crect compound mutants? Are these mice viable?
4. In Figure 1 and Figure 5 it is unclear how cell numbers were determined (number of apoptotic MES cells to total number of MES cells). How were cell boundaries determined?
5. The cell imaging results are not matched with molecular data, which is a missed opportunity. With emerging molecular detail in mouse and zebrafish models, scRNAseq studies, genetic mutant analysis of cleft models, it would be more informative to connect some of the cell behavior to the genes that regulate the process. The study visualized p63 and cadherin, both genes have been implicated in molecular regulation of palate development in model organisms and human CLP cohorts, but molecular or genetic analysis of this cell migration process are lacking here.
6. There is no comment on the morphology of the lip and primary palate in the Crect BAX model here. It would be surprising if broad epithelial deletion of BAX in periderm does not cause lip/facial or primary palate phenotypes. Please show data.
7. Crect expression is much broader than periderm and is variable among litters, or even pups within litters - how was this variability and lack or periderm specificity accounted for in the experiments? Recommend confirming results with another epithelial Cre driver?

In total, nice study, very focused on secondary palate, but the study should be placed in context of palatogenesis and could be more impactful if there was molecular detail to go along with the cell migration descriptions.

© 2022. Published by The Company of Biologists under the terms of the Creative Commons Attribution License (https://creativecommons.org/licenses/by/4.0/).

Minor Comments:

1. In figure 2 and figure 3, do the time point labels apply to the other non-labeled images in the panel? For example, in 2C are those also 0, 5, 10, 15 and 20h?

First revision

Author response to reviewers' comments

We thank the reviewers for their time and detailed reading of our manuscript and for their enthusiasm for our findings. We are also grateful for their thoughtful suggestions and requests for clarification and further information. We have attempted to address all of the concerns of the reviewers by 1) performing extensive additional live imaging analysis and nuclear tracking analysis 2) Performing extensive additional quantification of apoptosis and cell migration 3) Improving visualization of data in figures 4) Including additional text providing context and more precise description of our findings. We think that these changes dramatically improve the manuscript and describe them in point-by-point responses below.

Reviewer 1
1.1. My main problem with this manuscript is that it would benefit of a more quantitative approach to address some of the open questions

We recognize this general perspective from the reviewer. We have performed extensive additional experiments and added substantial quantitation for numerous experiments, which we hope will address these concerns. Please see specific responses below.

1.2. Although the authors claim that epithelial cells are removed by collectively migrating towards the surface epithelium, such directionally is not so evident in the movies provided. In each stream there are cells with low persistence, and some even move away from the surface epithelium. In addition, the net movement of a whole stream or cluster seems that does not go in the direction of the surface epithelium (see for example the cell tracked in Fig 2E and corresponding movie: the bottom of the stream does not move upwards as expected)

We understand the reviewer's concern that the cell tracking data that we provided in previous Fig. 2E does not clearly capture the migration of epithelial trail cells. We have therefore performed an extensive set of additional live imaging experiments in order to perform nuclear tracking of epithelial cells during palate fusion using *Crect* $^{Tg/0}$; *R26*^{nTnG} embryos. In addition to tracking of individual nuclei, we have also now provided an additional analysis of nuclear movement which we present in two new figures (Fig. 3 and 4), and two new movies (Movie 5 and 6). Indeed, as the reviewer noted previously, not all cells migrate through trails with high persistence and we sometimes observe cells breaking away from trails and undergoing apoptosis, or remaining in islands through the course of our live imaging experiment (See Fig. 4; Movie 6). For both experiments, we have performed detailed cell tracking (Fig. 3C-Cvi; Fig. 4C-Cvi), and now additionally provide vector analysis to highlight collective directional migration (Fig. 3D; Fig. 4D). We have attempted to better describe this process in text that we have added in lines 213- 222.

1.3. How is the movement of epithelial clusters that are not directly connected with the surface epithelium? The example in Fig 6A suggest that between frames 10h and 20h the disconnected cluster become closer to the surface. The distance between each cell and the surface needs to be done to answer this kind of questions.

In our first submission we focused our migration studies on epithelial trails, and we would like to thank the reviewer for pointing out the need to also address epithelial clusters that are not directly connected with the surface epithelium (i.e. islands). As the majority of cells in islands distant from surface epithelium undergo apoptosis, we performed a set of additional live imaging experiments focusing on islands adjacent to but not connected to the surface

© 2022. Published by The Company of Biologists under the terms of the Creative Commons Attribution License (https://creativecommons.org/licenses/by/4.0/).

epithelium in *Crect* $^{Tg/0}$; *R26*^{nTnG} embryos. Consistent with what the reviewer noticed, our cell-tracking analysis revealed individual cells of the cluster migrating closer to and eventually merging into the surface epithelium (Fig. 4; Movie 6). These movements were akin to those seen from epithelial trail cells described above (Fig. 3; Movie 5), and there was much less apoptosis than seen in islands deep within the MES (Fig. 2D, Movie 4). This suggests that cells in epithelial islands adjacent to surface epithelium behave similarly to those of connected streams. We have attempted to describe this process in text that we have added in lines 218-222.

1.4. Considering the apparent low directionality of the moving cells, it is not clear how that could explain the almost complete removing of the epithelial with very little apoptosis. In my opinion extensive quantification of cell movement followed by a mathematical simulation could test the model put forward by the authors. In addition, a mathematical simulation could help to analyse the weight that different mechanisms (e.g., apoptosis, cell extrusion, cell migration) could have in epithelial fusion.

We agree with the reviewer's view that it is challenging to precisely disentangle the weight of different cell behaviors during fusion. To begin to test this question, we have performed additional cell tracking of epithelial trails as they undergo migration. (See also response to 1.2 above). We have also observed that *Bax*$^{lox/lox;}$ *Bak*$^{-/-}$; *Crect*$^{Tg/0}$ embryos exhibit thicker trails and longer retention of MES that may compensate for the absence of apoptosis. These data are added to Fig. S4J-L and described on lines 167-169 and 227-229. We are very interested in generating a mathematical simulation of these ideas in the future, but think that this is beyond the scope of the current work.

1.5. The authors show nicely that an actomyosin cable is formed at the edge of the epithelial clusters, and that global inhibition of myosin in epithelial cells impairs cell movement. Based on these experiments the authors conclude that the actomyosin cable is required for epithelial movement. However, this conclusion is premature, as the genetic inhibition of myosin performed in this manuscript could affect other actomyosin process different to the contraction of the actomyosin cable at the edge cluster. In order to test their hypothesis, the authors need to interfere specifically with the actomyosin cable at the edge cluster, which could be done by laser ablation or optogenetics.

We agree with the reviewer that highly localized manipulation of actomyosin cables will be required to determine exactly how actomyosin contributes to MES collective migration and are working to establish optogenetic methods to address these questions, but incorporating such methods into our ex vivo culture system is non-trivial. We have instead added text at lines 299-300 and 379-383 to make clear that we have not yet definitively proven whether actomyosin cables drive trail movement or whether actomyosin may only be required for collective organization of the MES during migration. We find it unlikely that actomyosin contractility will not be involved in both processes, and think a detailed characterization will be required.

1.6. Inhibition of myosin leads to single cell migration, suggesting that myosin is required to maintain cell-cell adhesion, as it has been previously published. In addition, these observations suggest that the reason why these single cells do not migrate correctly, is because they need to migrate collectively. This could be directly tested by blocking cell-cell junction in the epithelial cells, followed by analysis of migration.

We are very interested in untangling the specific roles of actomyosin contractility and cell adhesion in collective MES migration. As blocking cell-cell junctions through antibody blocking or calcium depletion would likely dramatically impact the integrity of the tissue, these studies will require careful spatiotemporal control. As described above, our lab is currently establishing optogenetic approaches for localized manipulations, but these fall outside of the scope of the current work. We have added text at lines 379-383 to make clear that our results are consistent with either migratory force generation or the maintenance of an epithelial collective.

© 2022. Published by The Company of Biologists under the terms of the Creative Commons Attribution License (https://creativecommons.org/licenses/by/4.0/).

1.7. The authors argue that although apoptosis plays a role in epithelial remotion, apoptosis inhibition does not affect removing of the epithelial at large. To sustain this apparently contradictory statement, the authors need to quantitatively compare epithelial behaviour with and without apoptosis. A possible explanation is that in absence of apoptosis alternative mechanism, such as migration, are stimulated to compensate for the lack of apoptosis removal. This need to be properly analysed.

Our new results indicate that epithelial trails in *Bax*$^{lox/lox;}$ *Bak*$^{-/-}$; *Crect*$^{Tg/0}$ embryos are thicker (Fig. 1K; Fig. S4J-L) and epithelium is retained longer in epithelial triangles, particularly in the posterior palate (Fig. 1M). Though we cannot definitively rule out additional compensatory mechanisms, these data are consistent with more MES cells being removed through collective migration.

Reviewer 2

2.1. Fig. 1A-C: It would be nice to see a mesenchymal cell stain (or at least DAPI) in addition to epithelial Ecad. It is difficult to discern whether the formation of these epithelial trails accompanies general changes in local cell density or whether the mesenchymal microenvironment appears fairly unchanged. The use of theterm “small epithelial holes appeared” (p. 5) implies these might be acellular areas, so it should be clarified if this is not the case.

We appreciate this point and recognize that from our previous description, it was not obvious what the epithelial trails were migrating through. We have added images including DAPI-stained nuclei in (Fig. 1B,E,H) and showing Vimentin-stained mesenchyme in (Fig. S1C). We have also added language to the text (lines 134-135) to emphasize that MES epithelial trails are surrounded by mesenchyme, and have changed the word epithelial “holes” to epithelial “breaks” to further clarify this point.

2.2. What is the proportion of mesenchymal cells that are cC3+? While it appears that most are epithelial, a greater number in the posterior palate appear to be mesenchymal from Fig. 1C. This becomes important later when the authors note that the Crect-Cre driver is active in posterior palatal mesenchyme (see related comments 4, 9).

We have now quantified apoptosis within volumes of mesenchyme generated from 30μm image stacks and find that it is extremely limited (Fig. S2G,H). From these results, we see a comparable amount of mesenchymal apoptosis along the anteroposterior axis. We do not have a reason to think that loss of apoptosis in the mesenchyme of the far posterior palatal shelf is contributing to delayed MES removal in this area, but we cannot rule it out with our current reagents and have added a statement in lines 165- 169 to express this possibility.

2.3. How much apoptosis is occurring within “trails” inside the MES as compared to epithelial “triangles” at the nasal and oral surfaces?

To quantify apoptosis within trails and epithelial triangles, we segmented the MES into four equal parts and considered the dorsal-most quarter to include nasal epithelial triangles, the next two quarters as the seam region containing epithelial trails and islands, and the ventral-most quarter to include the oral epithelial triangles (Fig. S2C, D). Apoptosis within E-cadherin-expressing regions within oral, nasal and middle segments was quantified. (See also Materials and Methods lines 486-502). As we describe in the results section (lines 140-145), though there is a greater absolute number of apoptotic cells in the epithelial triangles, normalization to epithelial volume shows that apoptosis in epithelial trails away from the oral and nasal surfaces was relatively greater than that observed in epithelial triangles (Fig. S2E-F).

2.4. The Crect-Cre mice used in this study (Reid et al, 2011) were previously characterized for their ability to act in craniofacial surface ectoderm beginning around ~E10.5. However, it is not clear when/where it is acting in oral epithelium during palate closure. From p.7 Results: “we did not observe any GFP reporter positive, E-cadherin negative cells throughout most of the palate,” this suggests it is epithelial-specific (at least anteriorly). However, this could be conclusively demonstrated using their mTmG reporter (Fig. S2) by showing the Tomato channel (where Cre is inactive) in addition to the GFP channel (where Cre is active). Similarly, in the Crect-Cre; R26nT/nG

© 2022. Published by The Company of Biologists under the terms of the Creative Commons Attribution License (https://creativecommons.org/licenses/by/4.0/).

experiments (Fig 2E,F), it would be very nice to be able to visualize the red (mesenchymal) nuclei as well as the green (epithelial) ones, which are shown.

We have now added additional characterization of Crect Cre-mediated recombination of the ROSA26^{mTmG} reporter in the secondary palate, which we show in Fig. S3 and describe in lines 149-153. These data include both the Tomato and GFP channels and reveal highly specific and efficient recombination only in the epithelium through most of the palate immediately before palatal shelf contact, and recombination within the palatal mesenchyme in only the far posterior of the palate (Fig. S3D,H).

We have now replaced experiments previously presented in Fig. 2E,F with those now in Fig. 3 and Fig. 4. Though these embryos harbor the R26^{nTnG} allele, and we agree that including the Tomato channel would be an aesthetic improvement, we elected not to image this channel as it substantially decreases viability of the tissue in these live imaging experiments.

2.5. It is curious that in Crect-Cre; Baxfl/fl; Bak-/- mice, apoptosis by both TUNEL and cC3 appears to be completely absent, even in the mesenchyme, although this is only quantified for Ecad+ cells

As described in response to comment 2.2, we have now quantified apoptosis in the mesenchyme and find that even in control embryos, there is very little apoptosis within mesenchymal cells surrounding the MES (Fig. S2G,H).

2.6. Bak-/- KO mice are used as the control group for most of the experiments. Is there any difference in the degree of apoptosis between these and WT mice?

We utilized *Bax*$^{lox/lox}$; *Bak*$^{-/-}$ mice as controls to enable the use of littermate controls in this complex genetic cross. We have quantified apoptosis in *Bax*$^{lox/lox}$; *Bak*$^{-/-}$ embryos in comparison with WT controls and find them to have comparable amounts of epithelial apoptosis (Fig. S4).

2.7. Palate closure in Crect-Cre Baxfl/fl; Bak-/- mutants is only investigated at E15.5, when it is concluded that PS fuse normally and “MES clearance complete[s] successfully,” but display abnormal migration. Previous studies of cell death mutants show that PS meet but do not fuse at E15.5, and global Bax; Bak Kos show a high penetrance (30%) of CP by E18.5. MES clearance is later ages should be investigated to determine is clefting occurs or may be delayed in Crect cKOs.

We have analyzed *Bax*$^{lox/lox}$; *Bak*$^{-/-}$; *Crect*$^{Tg/0}$ embryos at E17.5 and find complete closure of the secondary palate (n=3)(Fig. S4D-I). We have never observed a complete secondary palate cleft in more than 14 mutant embryos examined at E15.5.

2.8. Results, p. 8 “we generated a Krt6a iCre knock-in mouse line for tracking the migration of peridermal cells.” As far as I can tell, the method for generating this mouse is not described in the Methods section and no reference is provided. The term “iCre” is also not defined here and is ambiguous. Some groups use this term to refer to “inducible” (e.g. tamoxifen regulable CreER), while it can also mean “improved” (e.g., codon- optimized). I assume it is the latter here as no mention is made of tamoxifen regimens, but this should be clarified in the text.

We appreciate this request for clarification. The “i” of “iCre” indeed stands for “improved.” We have now provided more details on how this *K6a-iCre* line was generated, which we provide in the Materials in Methods on lines 417-430.

2.9. According to the text, only “mid-anterior MES” was imaged for the studies in Figs. 2/3. Given the unexpected finding that Crect-Cre is active in the posterior palatal mesenchyme, the authors should make use of this fortuitous finding to compare MES dissolution in the anterior and posterior palate region. Presumably all apoptosis (mesenchymal and epithelial) will be blocked in the posterior region of Crect-Cre; Baxfl/fl; Bak-/- mice while only epithelial apoptosis will be blocked in the anterior region.

As described in response to Comment 2.2, we find very little apoptosis within the palatal mesenchyme at any position (Fig. S2G,H), and therefore do not think that delayed removal of

© 2022. Published by The Company of Biologists under the terms of the Creative Commons Attribution License (https://creativecommons.org/licenses/by/4.0/).

the MES in the posterior palate is attributable to a loss of mesenchymal apoptosis. We cannot rule this out however, and we therefore mention this possibility in lines 165-169.

2.10. Results, p. 9: "p63-expressing basal epithelial cells undergo migration to the oral and nasal surface concomitant with the basal epithelial initiation of expression of periderm marker Krt6a." In my opinion, since I think the Krt6-iCre line used here is non-inducible, one must consider an alternative explanation, which is that migrating cells expressed Krt6a before initiating migration, rather than turning it on during the migration process. In fact, from the merged images in Fig. 4, it would appear that quite a few cells express either Krt6a or p63 heterogenously, as there are very few white (magenta + green) cells even in the trails. Again, here, I would love to see the red nuclei (Krt6a-iCre-negative) as well as the green. In any case, since there is a pretty strong precedent from the Dixon lab that periderm cells can migrate during MES dissolution, perhaps the authors could be more cautious in their conclusion and could perhaps preface this statement with "in addition to periderm, p63- expressing epithelial cells undergo migration…" Live imaging of the Krt6a-iCre; R26nTnG line could also unambiguously resolve whether Krt6a is turning on as cells migrate, though I concede this is a lot of work to ask for.

We appreciate this question. Our lineage tracing data in Fig. 6A (previously Fig. 4A) clearly show that prior to palatal shelf contact, *$Krt6a^{iCre}$; $R26^{nTnG}$* E14.0 embryos exhibit periderm-specific recombination that does not overlap with p63 expression. During MES migration at E15.0 (Fig. 6B-D), many cells express both p63 and the Krt6a-iCre lineage, which we have now annotated with arrows. Certainly, some p63+ Krt6a-iCre- and p63-,Krt6a-iCre+, can also be found, which we point out as well with arrowheads. We think our data are consistent with p63 expressing basal epithelia beginning to express Krt6a during MES migration, but we also agree that periderm cells that never expressed p63 migrate as well. We think that this is consistent with Dixon lab results and further specify this point in text in lines 343-345. Live imaging of *$Krt6a^{iCre}$; $R26^{nTnG}$* embryos would be very interesting, but we do not currently have a way to differentiate between cells that originate as periderm, or as p63-expressing basal epithelium. Future experiments will focus further on this question.

2.11. I don't believe it is explicitly stated in the text whether CP is ever observed in the Crect-Cre; Myh9fl/fl; Myh10fl/+ mice, though it is written that "epithelial inclusions persist" even at E17.5, which is reminiscent of the persistent MES phenotype previously described in Krt14-Cre; Myh9fl/fl and Tgfb3-Cre; Myh9fl/fl mice (Kim et al, 2015) by this group. I believe that this earlier paper only reported CP in Tgfb3-Cre; Myh9fl/fl; Myh10fl/fl double mutants, so what is not clear to me is whether the apparent lack of CP here is due to the residual copy of Myh10 or the use of the Crect-Cre driver. I am not sure why the authors excluded data on Crect-Cre; Myh9fl/fl; Myh10fl/fl double KO mice when they went to the trouble to examine Crect-Cre; Myh9fl/fl; Myh10fl/+ and Crect-Cre; Myh9fl/+; Myh10fl/fl genotypes.

In text added at line 273-274 we now make clear that *$Myh9^{lox/lox}$; $Myh10^{ox/+}$ $Crect^{Tg/0}$* embryos do not exhibit a cleft palate. Indeed, the reviewer is correct that we previously observed submucous cleft palate in the posterior of *$Myh9^{lox/lox}$; $Myh10^{lox/lox}$ $Tgf\beta3^{Cre/+}$*, but the this Cre mediates some recombination within the mesenchyme as well. We also previously observed a less severe failure to remove the secondary palate epithelium in *$Myh9^{lox/lox}$; K14-cre* consistent with incomplete epithelial recombination in this model. Use of the Crect mouse line was superior based on highly efficient recombination within the craniofacial ectoderm, however *$Myh9^{lox/lox}$; $Myh10^{lox/lox}$ $Crect^{Tg/0}$* embryos exhibit cardiovascular phenotypes and could not be recovered past E13.5 to evaluate secondary palate development. We now make mention of this fact in line 261-263.

2.12. There appears to be a discrepancy in the apoptotic behavior between anterior and posterior palatal regions reported in Figs. 1 and 5. In Fig. 5, there is a gradient of apoptosis that is higher in anterior and middle palate compared to posterior, in both WT controls (Myh9fl/fl; Myh10fl/fl) and Myh9/10 mutants. However, in Fig. 1D-E, there appear to be greater numbers of cC3+ cells in the posterior compared to anterior region at every age examined (E14.75-E15.5). Both examine cC3+ cells in different palate regions at E15.5 using the sagittal thick- sectioning technique, so I am not sure what can explain this dramatic difference between experiments.

© 2022. Published by The Company of Biologists under the terms of the Creative Commons Attribution License (https://creativecommons.org/licenses/by/4.0/).

We apologize for this confusion. We have added more description in the Methods section (lines 485-502) to help clarify. This discrepancy is due to differences in quantification methods performed between these figures (now Fig. 1 and 7). For quantifications for wild type Fig. 1, we show an apoptotic index that normalized the number of cC3$^+$Ecad$^+$ cells to the total number of MES (Ecad$^+$) cells (i.e. the percentage of dying MES cells). In *Myh9/10* mutants, E-cadherin was reduced and/or relocalized, complicating the use of its expression for accurate epithelial cell counting. We instead used its expression to generate a surface volume for normalizing the number of cC3$^+$Ecad$^+$ cells in *Myh9/10* mutants and controls presented in Fig. 7. We acknowledge that the volumes generated from E-cadherin expression in mutants are likely an under-estimation of the MES, given its reduced expression, and the effect size is therefore likely even larger than what we calculated. Because E-cadherin expression volume cannot reflect cell density, the apoptotic index shown in Fig. 1 cannot be directly compared to the amount of volume- normalized-apoptosis in Fig. 7.

2.13. When cC3 is shown in blue (e.g. Fig S2, Fig. 5), it is extremely difficult to see. I suggest showing single channel images in gray, or another color.

We appreciate this suggestion. We have made these changes and now include both monochrome individual channels and merged images in color for figures where this was an issue (now Fig. 7, Fig. S5).

Reviewer 3

3.1. Introduction immediately starts on discussing the secondary palate. To make the paper accessible to readers (scientists and clinicians), it is important to briefly make the following points. 1) the palate is made up of primary and secondary palate, there are bony and muscular portions, 2) functions of the palate and clinical relevance, 3) genetic pathways underlying primary palate and secondary formation are likely distinct with some overlaps and 4) mouse models focus on secondary palate. This context is important from the outset to frame the specific question being investigated here. I think most craniofacial biologists don't appreciate the significant differences of primary vs. secondary palate development. The Discussion should then take the data presented here and place it in the context of total palate development, how lessons from this epithelial collective migration apply, or not, to primary palate development, which is more clinically prevalent (CLP is observed more than CPO).

We appreciate this suggestion; we have added additional introductory text in lines 38-56 that distinguishes development of the secondary palate from development of the upper lip and primary palate.

3.2. There is no description of how the Krt6a iCre mouse was generated or validated. This is important since there is no consensus in the field on what marker truly labels the periderm and each mouse group seems to choose their favorite or what they have breeding. Consider citing Trevor Williams and characterization of the Crect line.

We have now provided more details on how this *K6a-iCre* line was generated and validated, which we provide in the Materials in Methods on lines 417-430. We have cited Reid et al., 2011 for the *Crect* mouse line and have additionally added an acknowledgement (line 510) to highlight the source of this valuable reagent.

3.3. What is the state of the palate in the Myh9/Myh10; Crect compound mutants? Are these mice viable?

Myh9$^{lox/lox}$*; *Myh10$^{lox/lox}$* *CrectTg/0 **compound mutant embryos could not be recovered alive after E13.5, thus precluding our ability to assess secondary palate development. We clarify this fact in text on line 260-262.**

3.4. In Figure 1 and Figure 5 it is unclear how cell numbers were determined (number of apoptotic MES cells to total number of MES cells). How were cell boundaries determined?

© 2022. Published by The Company of Biologists under the terms of the Creative Commons Attribution License (https://creativecommons.org/licenses/by/4.0/).

We apologize for this confusion in these figures (now Fig. 1 and 7), and please also see our response to comment 2.12. We have added more description in the Methods section (lines 486-502) to help clarify the differences in the quantification methods. For the wild-type samples quantified in Fig. 1, we were able to use the highly membrane-specific expression of E-cadherin to determine cell boundaries. We performed cell count analyses by examining cleaved caspase 3 and E-cadherin expression in 2D through a volumetric stack to determine cell boundaries. For *Myh9/10* mutant samples quantified in Fig. 7, we were unable to determine cell boundaries due to disrupted E-cadherin localization and signal level. Thus, for these samples and their corresponding controls, we instead used E-cadherin expression volume to approximate MES volume, though we acknowledge that this does not account for cell density and is likely an underrepresentation of the MES in mutants, which we describe in lines 268-270.

3.5. The cell imaging results are not matched with molecular data, which is a missed opportunity. With emerging molecular detail in mouse and zebrafish models, scRNAseq studies, genetic mutant analysis of cleft models, it would be more informative to connect some of the cell behavior to the genes that regulate the process. The study visualized p63 and cadherin, both genes have been implicated in molecular regulation of palate development in model organisms and human CLP cohorts, but molecular or genetic analysis of this cell migration process are lacking here.

We absolutely agree with the reviewer that this system is ripe for a related scRNA-seq and/or spatial transcriptomic analysis. To our knowledge, there has not been a scRNAseq study focused on secondary palate fusion. We acknowledge the abundance of open questions that remain; however, we think that an extensive molecular analysis is beyond the scope of the current study which seeks to define cellular mechanisms with detailed live imaging approaches.

3.6. There is no comment on the morphology of the lip and primary palate in the Crect BAX model here. It would be surprising if broad epithelial deletion of BAX in periderm does not cause lip/facial or primary palate phenotypes. Please show data.

Very interestingly and surprisingly, our initial data indicate that *$Bax^{lox/lox}$*; *$Bak^{-/-}$*; *$Crect^{Tg/0}$* embryos do not exhibit an overt cleft lip/primary palate phenotype. Because we found more subtle phenotypes in secondary palate fusion in these mice, and because of the historically controversial role of apoptosis in this process, we prefer to perform a detailed and careful analysis of whether any lip or primary palate phenotype can be observed, which we will report in a future publication.

3.7. Crect expression is much broader than periderm and is variable among litters, or even pups within litters - how was this variability and lack or periderm specificity accounted for in the experiments? Recommend confirming results with another epithelial Cre driver?

This is an excellent point, indeed, we also noted that *$Crect^{Tg/0}$* mediated sporadic ectopic activity in around 15% of embryos examined, but these embryos were discernible by ectopic reporter activity, and by the presence of vascular phenotypes. We report this caveat in lines 187-190 and do not think it impacts the conclusion of our paper. As this work required complex multi-generational crosses, confirming the results with another epithelial driver is not feasible, and frankly, despite this caveat, we do not think there is currently a better epithelial Cre driver for this purpose.

3.8. In figure 2 and figure 3, do the time point labels apply to the other non-labeled images in the panel? For example, in 2C are those also 0, 5, 10, 15 and 20h?

We apologize for this confusion. We have updated all timelapse figures to better label the timepoints displayed.

© 2022. Published by The Company of Biologists under the terms of the Creative Commons Attribution License (https://creativecommons.org/licenses/by/4.0/).

Second decision letter

MS ID#: DEVELOP/2021/200181

MS TITLE: A unique form of collective epithelial migration is crucial for tissue fusion in the secondary palate and can overcome loss of epithelial apoptosis

AUTHORS: Teng Teng, Camilla S Teng, Vesa Kaartinen, and Jeffrey O. Bush
ARTICLE TYPE: Research Article

I am happy to tell you that your manuscript has been accepted for publication in Development, pending our standard ethics checks.

Reviewer 1

Advance summary and potential significance to field

The authors have adequately addressed most of my previous comments

Comments for the author

The authors have adequately addressed most of my previous comments

Reviewer 2

Advance summary and potential significance to field

As I mentioned in my first comments, this is an outstanding paper which uses innovative imaging and genetic approaches to shed new light on the contributions of apoptosis and collective cell migration in palatogenesis. They authors have made substantial revisions and improvements following the suggestions of myself and the other two reviewers. They have more than adequately answered my questions and addressed my concerns. I am very supportive of publication in Development.

Comments for the author

No additional suggestions, all comments have been thoroughly addressed.

Reviewer 3

Advance summary and potential significance to field

MS ID#: DEVELOP/2021/200181 MS title: A unique form of collective epithelial migration is crucial for tissue fusion in the secondary palate and can overcome loss of epithelial apoptosis Authors: Teng Teng, Camilla S Teng, Vesa Kaartinen, and Jeffrey O. Bush Article type: Research Article

This study utilizes murine genetic models to study the embryonic epithelium of the midline epithelial seam (MES) important in fusion of palatal shelves of the secondary palate. Many prior studies have suggested that apoptosis plays a key role in MES dissolution to permit fusion of palatal shelves. This study carried out genetic deletion of apoptosis regulator BAX and BAK but found that formation of the MES was unperturbed. The epithelial cells residing in the MES was able to undergo morphological and differentiation changes to mediate palatal fusion without problems. Using static- and live-imaging approaches, the authors described that small breaks in the MES consolidate into an interconnected network of epithelial trails connecting to the oral and nasal surfaces, and epithelial islands that undergo apoptosis or migrate through the mesenchyme. Whereas adherens junctions couple epithelial trail cells during migration, filamentous actin is anisotropically enriched at the edges of trails. Actomyosin contractility is critical for this unique form of epithelial migration, and

© 2022. Published by The Company of Biologists under the terms of the Creative Commons Attribution License (https://creativecommons.org/licenses/by/4.0/).

its disruption results in the dissolution of epithelial collectives and failure to complete secondary palatal shelf fusion. These results taken in context of other emerging corroborating data from zebrafish and human genetics, point to the evolutionarily conserved role of periderm biology in orofacial cleft pathogenesis.

I have studied the itemized responses to reviewers 1 and 2 and believe that the authors have satisfactorily addressed concerns and the manuscript is improved and suitable for publication.

Comments for the author

no additional comments. very nice study.

© 2022. Published by The Company of Biologists under the terms of the Creative Commons Attribution License (https://creativecommons.org/licenses/by/4.0/).
